# Single-cell spatial transcriptomics of formalin-fixed, paraffin-embedded biopsies reveals colitis-associated cell networks

**DOI:** 10.1172/JCI202488

**Published:** 2026-06-09

**Authors:** Elvira Mennillo, Madison L. Lotstein, Gyehyun Lee, Julian H. Hou, Vrinda Johri, Donna E. Leet, Christina A. Ekstrand, Jessica Tsui, Jun Yan He, Uma Mahadevan, Walter Eckalbar, Ryan M. Gill, Christopher J. Bowman, David Y. Oh, Gabriela K. Fragiadakis, Michael G. Kattah, Alexis J. Combes

**Affiliations:** 1 Division of Gastroenterology and Hepatology, Department of Medicine, Mayo Clinic, Rochester, Minnesota, USA.; 2 Division of Gastroenterology, Department of Medicine,; 3 Biomedical Sciences Graduate Program,; 4 UCSF CoLabs Initiative,; 5 Division of Hematology/Oncology,; 6 UCSF Bakar ImmunoX Initiative,; 7 Division of Pulmonary, Critical Care, Allergy and Sleep Medicine, Department of Medicine,; 8 Department of Pathology, and; 9 Division of Rheumatology, UCSF, San Francisco, California, USA.

**Keywords:** Gastroenterology, Inflammation, Inflammatory bowel disease, Transcriptomics

## Abstract

Imaging-based, single-cell, spatial transcriptomics (iSCST) of FFPE tissue enables comprehensive analysis of archived specimens while preserving spatial context, critical to an understanding of ulcerative colitis (UC) pathology. Here, we deployed a robust framework for applying iSCST to clinical FFPE mucosal biopsies from patients with UC or immune checkpoint inhibitor–induced colitis, as well as patients serving as healthy controls. iSCST using custom Xenium gene panels enabled precise detection of diverse cell subsets and disease-specific genes. We mapped transcriptionally distinct fibroblast subsets within mucosal niches, including inflammation-associated fibroblasts (IAFs), and identified colitis-specific neighborhoods formed by IAFs, monocytes, and neutrophils. Transcriptional signatures and spatial neighborhoods uncovered through iSCST were associated with vedolizumab (VDZ) response, with nonresponders exhibiting either an innate IAF-monocyte-neutrophil signature or adaptive gut-associated lymphoid tissue signature, while responders showed enrichment of an epithelial cellular neighborhood. These signatures were validated in an internal and an external dataset, supporting the existence of 2 distinct archetypes of treatment resistance to VDZ in UC. This iSCST framework provides a powerful approach for analyzing FFPE tissues, offering insights into colitis-associated cellular networks and identifying biomarkers to enhance patient risk stratification in routine clinical workflows.

## Abstract 2

We applied spatial gene mapping to archived colitis biopsies, identifying immune cell neighborhoods that explain why some patients resist vedolizumab and others respond well

## Introduction

Single-cell multiomics studies in inflammatory bowel disease (IBD) have rapidly accelerated our understanding of intestinal inflammation, highlighting critical roles for multiple cell subsets across stromal, epithelial, and immune compartments (1–8). While powerful, traditional single-cell multiomics analyses of prospectively collected and digested colon biopsies suffer from biased cellular dropout (leading to underrepresentation of epithelial and granulocyte subsets), loss of spatial information, and prolonged patient recruitment (9, 10). Despite extensive publicly available multiomics data on IBD, precision medicine approaches from tissue are still nascent and unrealized, at least in part due to the special collections required for cryopreservation or bulk RNA-seq, which are not part of clinical standard of care. Image-based, single-cell, spatial transcriptomics (iSCST) of FFPE tissue with subcellular resolution directly addresses these shortcomings by comprehensively capturing cell subsets and transcriptional states in mucosal biopsies, retaining spatial relationships, and accelerating recruitment for retrospective, longitudinal, case-control analyses of archived clinical specimens. A growing body of literature leverages iSCST to study mouse models (11) or tumor samples (10, 12–18), but few studies have applied an optimized iSCST approach to define cellular niches in ulcerative colitis (UC) using archived FFPE clinical specimens collected during routine standard of care.

Here, we applied iSCST technologies to UC samples to explore the spatially resolved gene signatures and tissue organization that define this pathology. We analyzed FFPE colon mucosal biopsies archived for up to 11 years from non-IBD controls, patients with UC, and patients with immune checkpoint inhibitor–induced (ICI-induced) colitis. To optimally extend iSCST to archived clinical specimens from inflammatory intestinal disorders, we compared 3 commercially available iSCST platforms capable of analyzing FFPE specimens with subcellular resolution from tissue sections and compared smaller focused gene panels to larger universal panels with broader transcriptomic coverage. Our custom UC panel identified transcriptionally and spatially distinct fibroblast subsets in health and disease, and we defined inflammation-associated fibroblast–myeloid (IAF-myeloid) and gut-associated lymphoid tissue (GALT) cellular networks that increased in abundance and spatial proximity in patients with active UC. We identify a gene set associated with responsiveness to vedolizumab (VDZ) in UC, validated in a replication iSCST dataset and a publicly available bulk transcriptomic dataset. The use of this optimized iSCST pipeline for analyzing archived mucosal biopsies from patients with inflammatory intestinal disorders elucidated key UC-associated cellular networks and treatment response signatures.

## Results

### iSCST approaches on archived FFPE mucosal colon biopsies from healthy and inflamed tissue.

**Figure 1:**
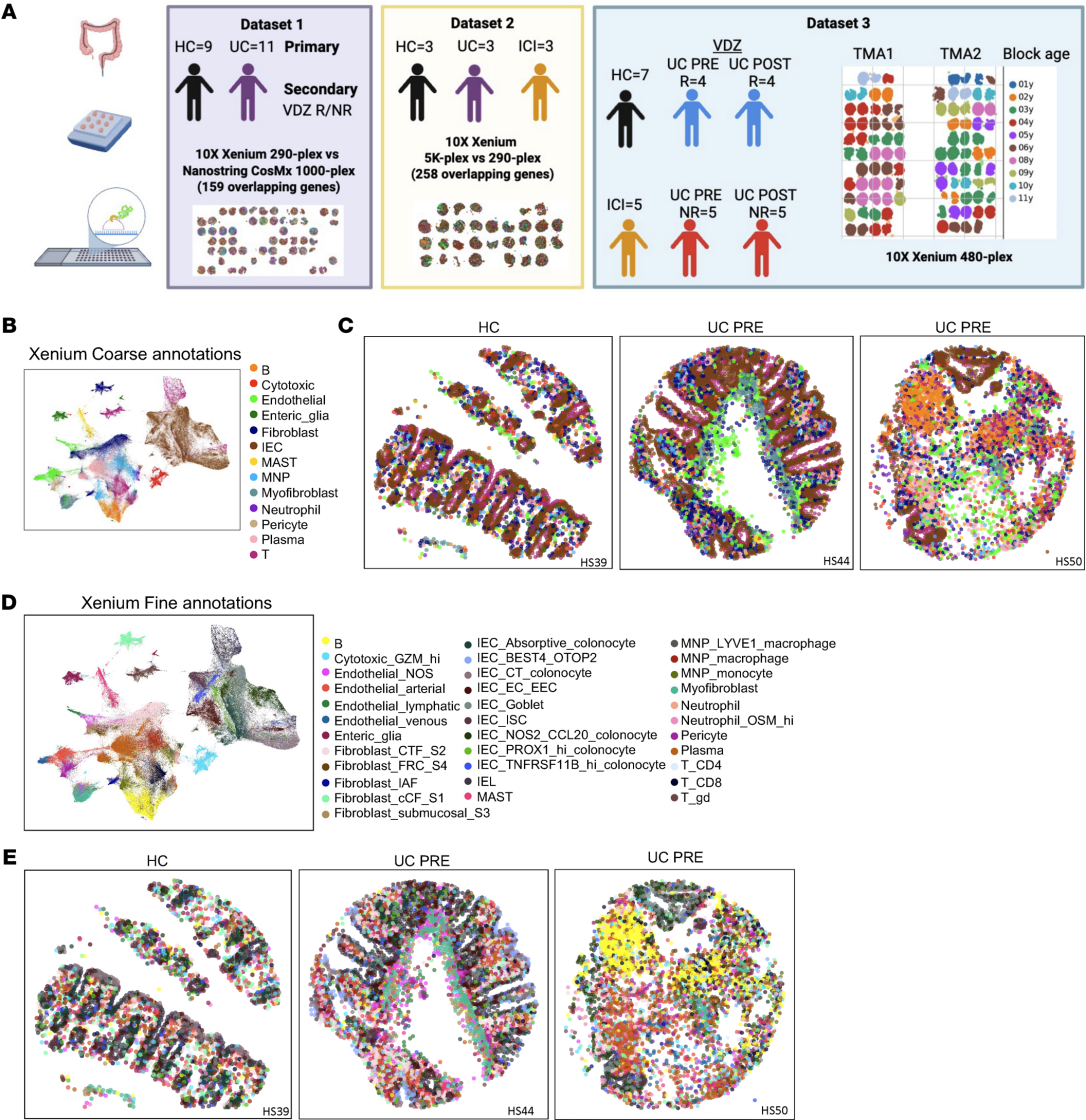
Study design and cell-type mapping in archived colon mucosal biopsies using imaging-based single-cell spatial transcriptomics. (**A**) Schematic of study design (created with BioRender). (**B** and **C**) UMAP (313,940 cells) (**B**) and representative spatial scatterplots (**C**) of Xenium Dataset 1 colored by coarse annotations. (**D** and **E**) UMAP (313,940 cells) (**D**) and representative spatial scatterplots (**E**) of Xenium Dataset 1 colored by fine annotations. The same cores are shown in **C** and **E**. MNP, mononuclear phagocyte; PRE, before VDZ treatment.

To establish an easily accessible and standardized pipeline, we focused our evaluation on 3 commercially available, FFPE-compatible platforms with subcellular resolution and whole-section imaging: Xenium (10x Genomics), CosMx (Bruker; formerly NanoString), and MERSCOPE (Vizgen). To reduce cost and minimize batch effects, we created a tissue microarray (TMA) comprising FFPE colon biopsies from patients with UC and those without IBD treated as healthy controls (HCs). This TMA was previously used by our group to generate single-cell spatial transcriptomic data using the 1,000-gene predesigned CosMx Human Universal Cell Characterization panel (9). The same TMA was then used to generate single-cell spatial transcriptomic data with Xenium (Figure 1) and MERSCOPE (Supplemental Figure 1; supplemental material available online with this article; https://doi.org/10.1172/JCI202488DS1). For these experiments, we designed custom panels containing 290 genes (Xenium) and 280 genes (MERSCOPE). Both panels were tailored to capture colon-associated mucosal and submucosal cell subsets as well as differentially expressed genes (DEGs) previously identified by single-cell or bulk transcriptomics of colon biopsies from HCs or patients with UC (1–10). The resulting data derived from this TMA are referred to as Dataset 1 (Figure 1A and Supplemental Table 1). Due to difficulties with tissue clearing, MERSCOPE could not detect most of the genes in the panel (Supplemental Figure 1, A and B), and only 0.17% of cells passed quality control (QC) (Supplemental Figure 1, C and D); therefore, MERSCOPE was excluded from the remainder of the analysis. To compare a focused custom Xenium 290-plex IBD panel to an expanded Xenium approximately 5,000-plex (5k) panel, we generated Dataset 2 using a second TMA comprising biopsies from HCs and patients with UC or ICI-induced colitis (Figure 1A and Supplemental Table 2). We further designed and implemented a custom Xenium 480-plex IBD panel to validate our findings from Dataset 1 in an independent group of patients containing biopsies from HCs and patients with UC or ICI-induced colitis (Dataset 3) (Figure 1A and Supplemental Table 3). Taken together, these 3 datasets and associated gene panels (Supplemental Table 4) constitute a clinically relevant collection of human mucosal colon biopsies spanning healthy tissue and 2 distinct colon-associated inflammatory disorders. This resource enables direct comparison of platforms, panel sizes, cell segmentation strategies, and FFPE block ages to inform the optimal approach for studying colitis.

### Enhanced sensitivity and cell-type resolution of FFPE colon mucosal biopsies using a customized Xenium gene panel.

To optimize a pipeline for the analysis of spatially resolved cellular networks in IBD, we performed a series of comparisons across platforms to assess segmentation, transcript and gene detection sensitivity, cell-type identification, and panel composition. We compared data generated from CosMx and Xenium in Dataset 1 according to different cell segmentation approaches. Although Xenium and CosMx used distinct segmentation methods in Dataset 1 — nuclear expansion (Xenium) and multimodal segmentation (CosMx) — their median cell areas were comparable, with only a few Xenium outliers exhibiting enlarged profiles (Supplemental Figure 2A). Xenium also showed a modestly higher fraction of non-nuclear transcripts compared with CosMx (Supplemental Figure 2B). Using Dataset 2, in which Xenium data were generated with multimodal cell segmentation, we resegmented the dataset using nuclear expansion to provide a direct comparison of the same images. Regardless of platform, the multimodal approach produced smaller cell areas than nuclear expansion while maintaining a similar fraction of nuclear transcripts (Supplemental Figure 2, C and D). Visualization of cell boundaries further showed that multimodal segmentation yielded tightly defined borders around nuclei compared with nuclear expansion, and this was most apparent in colonic crypts (Supplemental Figure 2, E and F).

Next, we evaluated transcript and gene detection sensitivity across iSCST platforms. The larger CosMx panel yielded a higher number of transcripts and unique features per cell (Supplemental Figure 3, A and B). However, when analysis was restricted to the 159 overlapping genes between panels, Xenium detected significantly more transcripts and unique features per cell (Supplemental Figure 3, A and B), as well as consistently higher transcript counts per gene (Supplemental Figure 3C). Xenium also exhibited a 10-fold lower detection of negative probes per cell (0.03 vs. 0.37; *P* < 0.001) (Supplemental Figure 3D). Together, the higher signal per gene with reduced nonspecific detection of negative probes produced a higher signal over background for Xenium compared with CosMx (Supplemental Table 5).

Next, we assessed how each platform’s specificity and sensitivity affected unsupervised cell-type identification in FFPE colon biopsies. Using the same processing pipeline, we generated UMAP embeddings, clustering, and cell-type annotations at both coarse (Figure 1, B and C, and Supplemental Figure 3E) and fine levels (Figure 1, D and E, and Supplemental Figure 3F). Compared with the CosMx reanalysis (9), results obtained with Xenium from the same TMA resolved a broader spectrum of colon-associated cell types, including fine epithelial, fibroblast, endothelial, and innate immune subsets such as neutrophils and mast cells (Figure 1, D and E). Because of the lower resolution of the CosMx data, subsequent analyses focused on coarse cell-type identification for direct comparison. Even at this coarse level, Xenium’s higher sensitivity yielded sharper gene expression signatures and clearer cluster separation (Supplemental Figure 3, G and H), whereas CosMx showed >20% unassigned cells due to indistinct profiles and a small “ncount_hi” population driven by high immunoglobulin gene expression (Supplemental Figure 3, I–K). Xenium allowed us to identify spatially and transcriptionally distinct fibroblast populations. Previous work by our group and others has highlighted the importance of distinct fibroblast subsets, characterized by unique transcriptional states, in colon tissue under steady-state conditions, as well as in UC pathology and treatment response (1, 2, 4, 6–10, 19). While the spatial location of those transcriptomic subsets has been recently described in IBD mouse models (11), equivalent detailed spatial characterization in clinical biopsies has been challenging and is still lacking. By leveraging the high sensitivity of our optimized iSCST approach, unsupervised clustering identified 5 transcriptionally distinct fibroblast subsets (S1, S2, S3, S4, and IAF) that were previously characterized in scRNA-seq of human colonic stromal cells (1, 9, 19). In agreement with prior scRNA-seq studies, Xenium iSCST identified transcriptionally distinct S1 (*CCL8*, *ADAMDEC1*, *APOE*, *FABP5*), S2 (*F3*, *SOX6*, *PDGFRA*, *WNT5A*, *POSTN*, *CXCL14*), S3 (*GSN*, *OGN*, *CCDC80*, *C7*), S4 (*C3*, *TNFSF13B*, *IRF8*, *PTGDS*), and IAF (*IL1R1*, *TIMP1*, *CD44*, *IL13RA2*, *MMP1*, *MMP3*, *OSMR*, *NFKBIA*, *TNFAIP3*) subsets (Supplemental Figure 4A). These subsets display a broad range of functions, and some have previously been linked to specific locations in the colon (20). S1 fibroblasts have been linked to *PDGFRA^lo^* colonic crypt fibroblasts (cCFs) at the bottom of the colon crypts, and S2 fibroblasts have been described to line the colonic crypt and share features with *PDGFRA^hi^* crypt top fibroblasts (CTFs) that are also high in *SOX6* and *WNT5A* (1, 9, 19, 20). While IAF are disease associated, S1–S4 subsets were present at steady state. We spatially mapped the transcriptomic states present in colon tissue (Supplemental Figure 4B). Spatial scatterplots showed that the S1-cCFs were somewhat dispersed but biased more toward the crypt base, while S2-CTFs lined the colonic crypt, as previously described (Supplemental Figure 4B) (1, 9, 19, 20). S3 fibroblasts localize to the submucosa (Supplemental Figure 4B), while S4 fibroblasts express fibroblastic reticular cell–associated genes, including *TNFSF13B* or B cell activating factor and colocalize with B cells in GALT aggregates (Supplemental Figure 4B).

All iSCST technologies we evaluated are targeted approaches, making panel design a critical step. Thus, we next sought to evaluate how gene panel size influences data quality and interpretability. To this end, we leveraged the TMA of Dataset 2, which was profiled on the Xenium platform using both a 290-gene custom panel and a large 4,737-gene panel comprising 4,625 predesigned genes plus 112 custom additions (Supplemental Figure 5A). The 2 panels included 258 overlapping genes (Supplemental Table 4), enabling a direct comparison of panel size effects on data performance. Analysis of the expanded 5k Xenium panel confirmed that larger panels capture more total transcripts and unique features per cell (Supplemental Figure 5, B and C). However, when restricted to the 258 overlapping genes, the 290-gene custom panel outperformed the larger panel, showing higher mean transcript and feature counts per cell (Supplemental Figure 5, B and C), stronger per-gene expression (Supplemental Figure 5D), and finer cell-type annotation (Supplemental Figure 5, E–G). This can be explained by the number of probes per gene in each panel, which was a median of 8 for the 290-plex compared with 5k panels with a median of 3 probes per gene.

In sum, our deep characterization for iSCST platforms for FFPE showed that cell segmentation strategies have only a modest impact on transcript assignment for lineage-specific genes, with the multimodal cell segmentation yielding smaller and more consistent cell boundaries, eliminating the small percentage of enlarged cells produced by nuclear expansion. Among platforms, Xenium provided the highest transcript and gene sensitivity for studying colitis, enabling the detection of a broader spectrum of colon-associated cell types and transcriptional states, including those often lost during tissue dissociation such as neutrophils or enteric glia. This study also provides a comprehensive spatial map of transcriptomic subsets of colonic fibroblasts at steady state in archived clinical FFPE mucosal biopsies. While the 5k panel can enable relatively fine annotation with a more tissue and disease agnostic approach, these results indicate that smaller disease- and tissue-specific panels offer optimal and robust performance for iSCST profiling of FFPE colon mucosal biopsies under both homeostatic and pathologic conditions.

### Distinct spatial transcriptomic profiles of B cells, epithelial cells, fibroblasts, and myeloid subsets differentiate cases and controls in UC FFPE colon biopsies.

**Figure 2:**
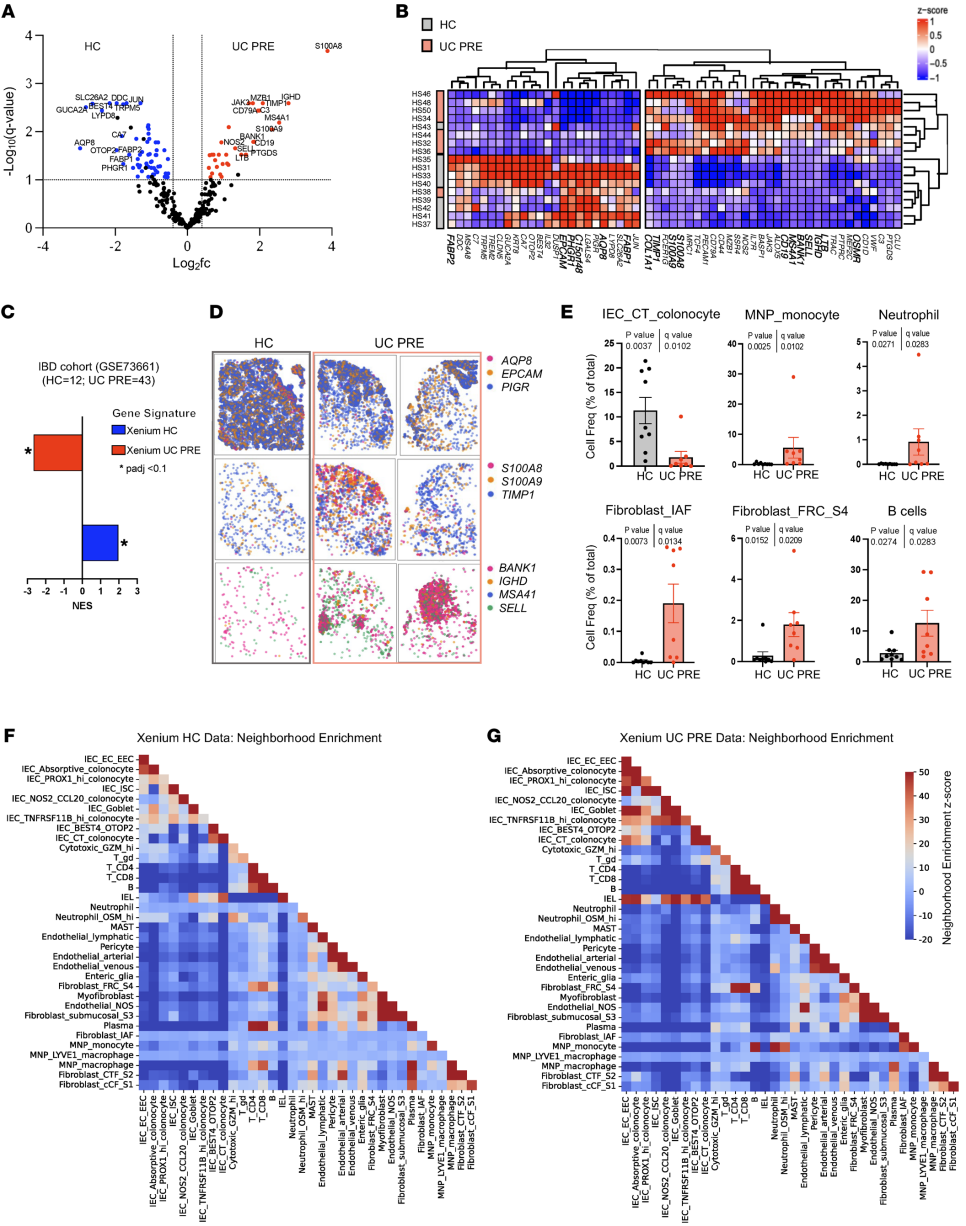
Disease-associated gene signatures and multicellular neighborhood in UC and validation in external data. (**A**) Volcano plot of pseudobulk DEG identified by DESeq2 with log_2_FC > 0.4 or < –0.4 and *q* < 0.1 in HC and UC PRE. (**B**) Heatmap of expression *z* scores for the indicated genes in UC PRE (up/down) relative to HC. (**C**) GSEA of Xenium HC and UC PRE spatial gene signatures in an external cohort of patients and relative NES, with significance determined by adjusted *P* value (*Padj) < 0.1. (**D**) Representative spatial transcript scatterplots highlighting a subset of genes relatively increased in HC and UC. (**E**) Cell frequencies of selected subsets comparing HC and UC PRE; each dot represents 1 patient (data presented as mean ± SEM); 2-sided Mann-Whitney test with FDR correction; *q* < 0.1 threshold for discovery; exact *P* and *q* values are shown. (**F** and **G**) Heatmaps displaying neighborhood enrichment *z* scores for fine annotation cell pairs within HC (**F**) and UC PRE (**G**) biopsies. For **B**, *z* score set at –1 to 1 for visualization purposes.

Next, we expanded our study beyond cell and subset identification to assess UC-associated transcriptomic signatures, focusing on the Xenium dataset while continuing parallel comparisons with CosMx on Dataset 1. To facilitate validation with bulk transcriptomic data, we generated pseudobulk iSCST profiles by aggregating transcript counts per patient and performed differential gene expression analysis comparing UC samples before VDZ treatment (UC PRE) to HCs across both platforms. Xenium identified 32 genes upregulated in UC PRE and 60 in HCs (Figure 2A and Supplemental Figure 6A), and unsupervised clustering of top DEGs clearly separated UC PRE and HC samples (Figure 2B). In contrast, CosMx identified 57 genes upregulated in UC PRE and only 1 in HCs (Supplemental Figure 6, A and B). GSEA using an external bulk transcriptomic dataset (21) confirmed significant enrichment of Xenium-derived UC PRE and HC signatures (Figure 2C), with a slightly higher normalized enrichment score (NES) for Xenium than CosMx (−2.66 vs. −2.49; Supplemental Figure 6C). While both platforms showed concordance with bulk data, CosMx exhibited greater discordance, inappropriately identifying 13 intestinal epithelial cell–associated (IEC-associated) transcripts (*KRT20*, *LGALS3*) as being upregulated in UC, when Xenium and external data both identified these transcripts as increased in HC biopsies with intact epithelium (Supplemental Figure 6D and Supplemental Tables 6 and 7). This finding indicates that Xenium exhibits better alignment with external bulk RNA-seq data. Therefore, we focused our subsequent spatial analysis of colitis using the Xenium datasets.

UC-associated DEGs from the Xenium dataset included genes characteristic of inflammatory infiltrates: *S100A8/S100A9* (neutrophils and monocytes); *CD19*, *LTB*, *SELL*, *MS4A1*, and *BANK1* (lymphocytes); and *OSMR*, *TIMP1*, and *COL1A1* (fibroblast markers), with cell-type identities confirmed in the Xenium iSCST data (Figure 2, A and B, and Supplemental Figure 6E). Conversely, HC-associated genes were predominantly linked to intact IECs, including *C15orf48*, *EPCAM*, *AQP8*, *FABP1*, *FABP2*, and *PHGR1* (Supplemental Figure 6E). We next leveraged the spatial coordinates of individual transcripts to generate spatial scatterplots for DEGs identified by Xenium in HC and UC PRE biopsies. IEC-specific genes (*AQP8*, *EPCAM*, *PIGR*) were enriched in HC tissue, whereas B cell genes (*BANK1*, *IGHD*, *MS4A1*, *SELL*) and myeloid markers (*S100A8*, *S100A9*) were increased in UC PRE samples (Figure 2D). Spatially resolved Xenium iSCST on Dataset 1 identified 34 transcriptomic cell subsets, including fragile populations such as neutrophils that are typically lost during dissociation. This enabled analysis of cell composition, gene expression, and spatial organization in intact colon tissue without cellular dropout. Comparing HC and UC PRE samples revealed 19 cell subsets with significantly altered abundance, including increased monocytes, neutrophils, fibroblast subsets (IAF and S4), endothelia, pericytes, B cells, plasma, and T cell subsets, and a reduction in IECs (Figure 2E and Supplemental Figure 7A).

Finally, to investigate spatial organization, we constructed neighborhood graphs for HC and UC PRE tissues and calculated enrichment *z* scores for all cell-type pairs (Figure 2, F and G). UC PRE tissue displayed increased proximity between intraepithelial lymphocytes and epithelial cells, as reported in murine colitis (11), and greater spatial association of monocytes with IAFs, neutrophils, and B cells (Figure 2G). Overall, UC FFPE biopsies exhibited a transition from an epithelium-dominated architecture in HC to spatially organized immune-stromal neighborhoods in UC, characterized by enriched B cells, myeloid cells, and fibroblast subsets.

### IAF-monocyte-neutrophil hubs distinguish UC from ICI-induced colitis.

**Figure 3:**
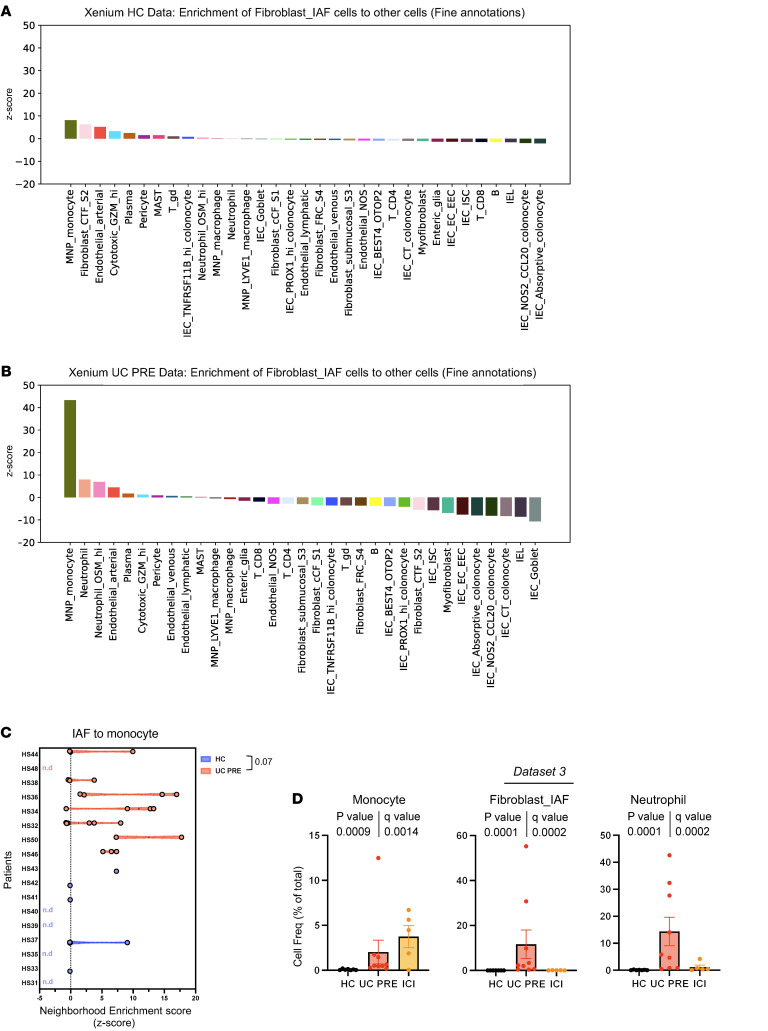
Neighborhood analysis reveals IAF-myeloid cell hubs that distinguish UC from ICI-induced colitis. (**A** and **B**) Spatial enrichment of IAF cells to all other cell types in HC (**A**) and UC PRE (**B**) biopsies. (**C**) Violin plots comparing the spatial enrichment of IAFs and monocytes by patient; each dot represents a core; 2-sided Mann-Whitney test. nd, not defined. (**D**) Cell frequencies of selected subsets comparing HC, UC PRE, and ICI conditions in Dataset 3; each dot represents 1 patient (data presented as mean ± SEM). Kruskal-Wallis, Dunn’s multiple-comparison test with FDR correction; *q* < 0.1 threshold for discovery; exact *P* and *q* values are shown.

Given the ability of iSCST to precisely detect rare fibroblast transcriptomic subsets in situ in human tissue (Supplemental Figure 5), including the IAF subset previously identified as a key driver of UC pathogenesis and strongly enriched in UC patients (Figure 2E and Supplemental Figure 7B), we leveraged our spatial transcriptomic dataset to characterize the composition of the IAF-associated cellular neighborhood. We focused on neighborhood enrichment *z* scores between IAFs and all other cell types in HC (Figure 3A) and UC PRE (Figure 3B). Monocytes exhibited the highest interaction scores in both HC and UC PRE, with a collectively higher value in UC PRE. The IAF-monocyte enrichment was followed by IAF-neutrophil enrichment, which has been described in recent single-cell analyses of UC biopsy specimens (15, 18). To corroborate and refine these transcriptional distinctions, monocytes were characterized by elevated expression of *HLA-DRA*, *HLA-DRB1*, and *VCAN*, while neutrophils exhibited higher levels of *CSF3R*, *FCGR3B*, *S100A8*, *S100A9*, and *TREM1*, thereby confirming the resolution of these 2 myeloid subsets (Supplemental Figure 7C). To investigate this interaction further, we calculated the neighborhood enrichment *z* scores between IAF and monocytes for each individual core and averaged the scores per patient. This revealed low enrichment scores in HC biopsies, with multiple instances where *z* scores could not be calculated due to low or no abundance (Figure 3C). UC PRE biopsies showed a trend (*P* value 0.07) toward increased *z* scores for IAFs and monocytes, consistent with the increased abundance and proximity of IAFs and monocytes in colitis. Using Dataset 3, which included both UC and ICI-induced colitis samples, we assessed whether the IAF-monocyte network identified in UC was also present in ICI colitis. Comparison of cell abundances across HC, UC PRE, and ICI colitis groups confirmed increased monocytes, neutrophils, and IAFs, along with reduced epithelial subsets, in UC PRE compared with HC (Figure 3D and Supplemental Figure 8A). Notably, the expansion of neutrophils and IAF was restricted to UC PRE and absent in ICI colitis, suggesting that this inflammatory stromal-myeloid niche may be specific to UC. Additionally, neighborhood enrichment analysis in ICI patients showed stronger spatial interaction of monocytes with macrophages, CD8^+^ cytotoxic T cells, Tregs, and neutrophils (Supplemental Figure 8B). Pseudobulk differential expression analysis comparing ICI colitis versus UC PRE and ICI colitis versus HC revealed 52 genes upregulated in ICI colitis and 81 in UC PRE (Supplemental Figure 8C and Supplemental Tables 8 and 9). ICI colitis exhibited more cytotoxic and resident memory T cell–associated genes, including *CD8A*, *GZMA*, *CCL5*, and *ITGAE*, confirming a distinct inflammatory profile for these 2 disease states, as previously described (22–24). Among the top genes enriched in UC PRE were *MMP1*, *MMP3*, *IL1B*, *OSM*, *S100A8*, and *CSF3R*, all associated with IAFs, monocytes, and neutrophils (Supplemental Table 9). Together, these findings define an IAF-monocyte-neutrophil cellular hub that is increased in abundance and spatial proximity in active moderate-to-severe UC, but is not a defining feature of ICI colitis, highlighting UC-specific inflammatory architecture.

### Spatial gene signatures associated with responsiveness to VDZ before treatment.

**Figure 4:**
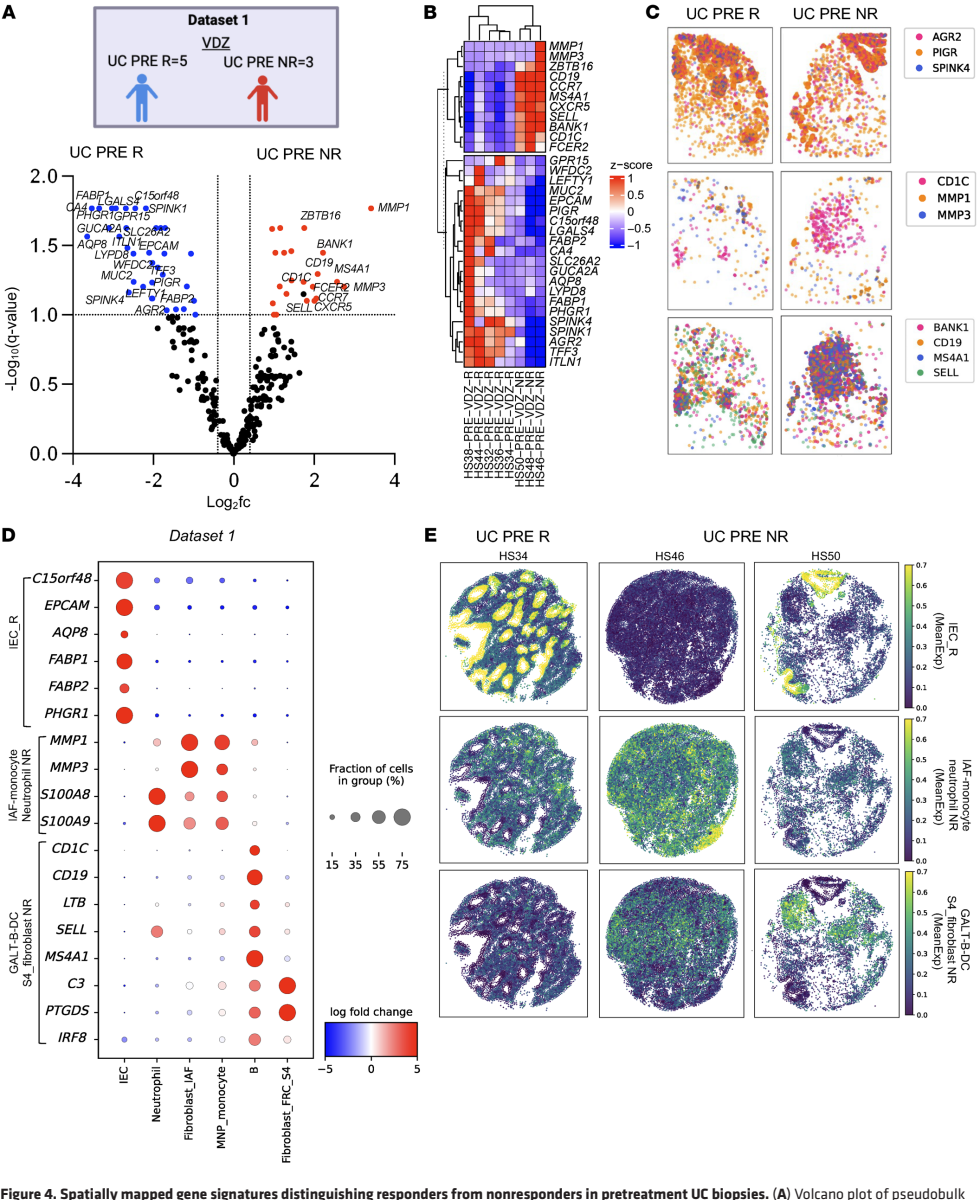
Spatially mapped gene signatures distinguishing responders from nonresponders in pretreatment UC biopsies. (**A**) Volcano plot of pseudobulk DEGs identified by DESeq2 with log_2_FC > 0.4 or < –0.4 and *q* < 0.1 in UC PRE NR versus UC PRE R. (**B**) Heatmap of expression *z* scores for the indicated genes in UC PRE NR (up/down) relative to UC PRE R. (**C**) Representative spatial transcript scatterplots highlighting a subset of genes relatively increased in UC PRE R (left) and UC PRE NR (right). The fields of view shown here were also used in Figure 2D. (**D**) Dot plot representation of selected signature genes for the indicated subsets. (**E**) Spatial scatterplot of representative cores for UC PRE R and UC PRE NR pseudocolored by mean gene signature expression per cell. The HS50 core shown here was also used in Figure 1, C and E. For **B**, *z* score set at –1 to 1 for visualization purposes.

Finally, to assess the association of spatial cellular networks with treatment outcomes, pretreatment samples were stratified by the patient’s subsequent treatment response as responders (UC PRE R) and nonresponders (UC PRE NR) to VDZ. We first conducted a pseudobulk DEG analysis between UC PRE R and UC PRE NR (Figure 4A and Supplemental Table 10). Consistent with previous findings (9), Dataset 1 identified response-associated genes linked to IEC crypts, including *AGR2*, *PIGR*, and *SPINK4*. Additionally, we observed an increase in IAF and myeloid-associated genes, such as MMP1 and MMP3 for IAF and FCER2 and CD1C for myeloid-associated genes in nonresponders (Figure 4, A–C). Moreover, UC PRE NR biopsies were enriched for genes associated with B cells (*CD19*, *MS4A1*, *BANK1*, and *SELL*) compared with UC PRE R (Figure 4, A–C). These B cell genes were located spatially in GALT aggregates (Figure 4C). This raised the possibility that some VDZ nonresponders have higher levels of GALT pretreatment compared with responders, supporting recent work showing that VDZ reduces GALT in responders after treatment (25). These data suggest the presence of at least 2 archetypes of nonresponder patients: an innate IAF-myeloid type and a chronic, adaptive, GALT-associated type. To test this hypothesis, we divided the Xenium VDZ nonresponse signature into 2 components: 1 comprising genes expressed by IAFs, monocytes, and neutrophils and 1 enriched in GALT aggregates, including landmark genes of B cells, dendritic cells, and S4 fibroblasts (Figure 4D and Supplemental Table 11). Together with the response signature (IEC_R), these gene modules were transcriptionally and spatially mapped at the single-cell level in Dataset 1 across UC PRE R and UC PRE NR cores. Spatial localization revealed IEC_R expression predominantly within crypt regions, whereas the 2 nonresponse signatures localized to IAF-monocyte-neutrophil clusters and GALT aggregates, respectively (Figure 4, D and E).

To further validate our gene signatures of response and nonresponse to VDZ identified in Dataset 1, we analyzed Dataset 3, consisting of archived FFPE mucosal biopsies collected before and after VDZ treatment, including samples preserved for up to 11 years. We performed Xenium analysis on Dataset 3 using a 480-plex customized panel, which included 275 genes overlapping with the previous 290-plex design. Because the FFPE blocks used ranged from 1 to 11 years after collection, we evaluated the effect of block age on Xenium data quality. Block age had only a marginal effect on the number of transcripts and unique features per cell when comparing block-level medians, with decreased performance noticeable primarily for samples ≥ 9 years old (Supplemental Figure 9, A and B). Despite the broad range of block ages, all samples passed QC, and some 11-year-old blocks performed comparably, or even better, than those aged 4–5 years. Analysis of coarse cell-type composition in UC samples grouped by block age (1–5 vs. 6–11 years; Supplemental Figure 9C) revealed no differences in cell-type proportions. Together, these results indicate that although block age modestly affects transcript and feature counts, it does not compromise overall data quality or cellular composition; thus, we proceeded with the full cohort for downstream validation analyses. Furthermore, all UC PRE biopsies from responders and nonresponders in Datasets 1 and 3 showed no significant differences in histologic score or endoscopic severity (Supplemental Figure 9D and Supplemental Tables 1 and 3), ensuring that the cellular and molecular differences identified through iSCST are not confounded by baseline clinical severity. Therefore, this cohort was well suited as a dataset to evaluate whether molecular and spatial signatures can capture pretreatment differences that gold standard endoscopic or histological assessments alone fail to detect.

**Figure 5:**
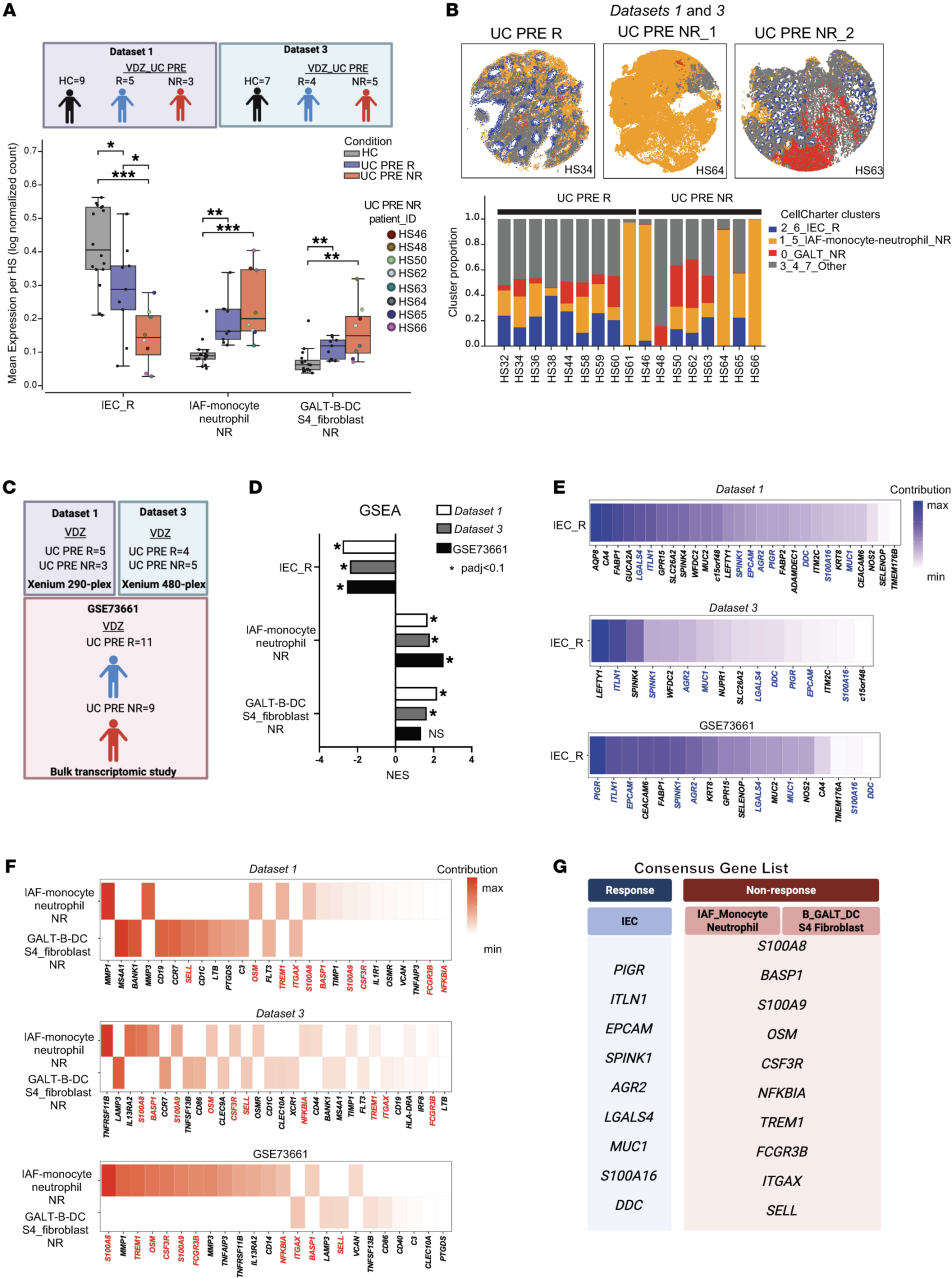
Cross-dataset validation of pretreatment response and nonresponse signatures and identification of a consensus gene list. (**A**) Schematic of the response datasets (top) (created with BioRender). Mean expression of each gene signature per patient across Datasets 1 and 3 in HC, UC PRE R, and UC PRE NR (bottom). The box-and-whisker plots depict the minimum and maximum values (whiskers), the upper and lower quartiles, and the median. (**B**) Spatial scatterplot of representative cores for UC PRE R and UC PRE NR pseudocolored by CellCharter spatial cluster annotation (top) and proportion of cells assigned to each annotated CellCharter cluster in UC PRE R and UC PRE NR (bottom). The HS34 core shown here was also used in Figure 4E. (**C**) Schematic of the 3 datasets used for validation (created with BioRender). (**D**) GSEA NES of Xenium signatures in 3 datasets comparing UC PRE NR and UC PRE R with significance determined by *Padj < 0.1 calculated using the Benjamini-Hochberg method. (**E** and **F**) Leading-edge genes in IEC_R (**E**), IAF-monocyte-neutrophil NR, and GALT-B-DC-S4_fibroblast NR signatures (**F**) across different datasets; consensus genes across datasets are labeled in blue (**E**) and red (**F**). (**G**) List of consensus genes associated with treatment responsiveness. For **A**, pairwise 2-sided Mann-Whitney *U* tests were performed between groups within each gene set, with Benjamini-Hochberg FDR correction applied across all comparisons; *Padj < 0.05, **Padj < 0.01, and ***Padj < 0.001.

Using Dataset 3, we confirmed both responder- and nonresponder-associated transcriptomic signatures within UC PRE-R and UC PRE-NR cores. Gene signatures were evaluated in our datasets, including HC (*n* = 16), UC PRE R (*n* = 9), and UC PRE NR (*n* = 8), by calculating the mean expression of the 3 gene signatures for each patient (Figure 5A). Next, we sought to assess how these distinct response signatures impact spatial neighborhood organization in pretreatment mucosal biopsies. To this end, we performed CellCharter spatial neighborhood analysis on the combined Datasets 1 and 3 to compare spatial neighborhood composition between UC PRE-R and -NR patients (26). This analysis identified 8 distinct spatial neighborhoods defined by varying cell-type compositions. Notably, several of these neighborhoods were distinctly enriched in the cell types expressing our response signatures: specifically, 2 neighborhoods were enriched in IEC, 2 were enriched in IAF-monocyte-neutrophil, and 1 was enriched in cells associated with the GALT signature (Supplemental Figure 10A). Focusing on neighborhoods enriched in our transcriptomic signatures, we confirmed that the IEC neighborhood is significantly enriched in responders, while nonresponders segregated into 2 distinct categories: 1 characterized by an innate IAF-monocyte-neutrophil neighborhood and 1 by an adaptive GALT neighborhood (Figure 5B and Supplemental Figure 10, B and C). These findings further support the existence of 2 distinct archetypes of nonresponders, highlighting the heterogeneity of treatment resistance to VDZ in UC.

We next performed GSEA of the 3 identified signatures across all UC PRE R and UC PRE NR from Datasets 1 and 3, as well as an external bulk transcriptomic dataset (GSE73661) to evaluate their reproducibility across independent cohorts and transcriptomic platforms (Figure 5C). GSEA consistently validated our Xenium VDZ response signature (IEC_R), which predominantly comprised epithelial genes (Figure 5D), across all 3 datasets. The IAF-monocyte-neutrophil signature was significantly enriched in VDZ nonresponders in both internal and external datasets. In contrast, the GALT-B-DC-S4 fibroblast signature was validated in Datasets 1 and 3 but did not reach statistical significance in the external dataset, again suggesting potential heterogeneity among VDZ nonresponders. To examine our gene signature more closely across datasets, we identified leading-edge genes from each GSEA and defined the genes within each gene set that contributed most significantly to the enrichment score (Figure 5, E and F). We next defined a consensus gene list by identifying overlapping genes consistently detected across all 3 datasets (Figure 5G), yielding a set of 9 responder-associated and 10 nonresponder-associated genes that capture both archetypes of treatment nonresponse in VDZ therapy. Collectively, using iSCST applied to retrospective FFPE cohorts, we defined a 9-gene pretreatment epithelial-enriched response signature and a 10-gene nonresponder signature that discriminate responders from nonresponders, highlighting distinct cellular archetypes underlying resistance to VDZ therapy in UC.

## Discussion

In this study, we defined spatial niches in UC, contrasting with HCs and patients with ICI-induced colitis, and identified gene sets and spatial cellular networks associated with colitis and VDZ responsiveness using clinical archived FFPE specimens. To establish a robust workflow for analyzing archived FFPE mucosal biopsies, we compared 3 commercially available platforms using the same FFPE TMA. For our goal of using archived FFPE to identify potential biomarkers and nominate pathways associated with treatment responsiveness, Xenium provided the most informative spatial datasets. The smaller custom Xenium panel, optimized here for mucosal biopsies of patients with IBD, improved cell state resolution across disease states and best captured the tissue features that define colitis. Spatial transcriptomics preserves cell subset frequency and transcriptional states with high fidelity, and these methods can facilitate robust tissue profiling using archived FFPE. Using this optimized spatial pipeline, we mapped transcriptionally distinct fibroblast subsets to discrete spatial locations and identified increased abundance and trends toward increased proximity of IAFs, monocytes, and neutrophils in patients with UC. Interestingly, this pattern was less prominent in a small cohort of patients with ICI-induced colitis, where monocytes and T cell–associated genes were increased, with the caveat that the UC biopsies were endoscopically more inflamed. We also identified spatial and transcriptional signatures associated with VDZ responsiveness, with nonresponders partitioning into 2 distinct archetypes consisting of either an innate IAF-monocyte-neutrophil signature or an adaptive GALT-associated signature, whereas responders were characterized by epithelium-associated gene programs. Leveraging an internal spatial validation dataset and a publicly available orthogonal bulk transcriptomic dataset, we identified a small consensus gene set across the 3 datasets with potential to risk-stratify patients prior to treatment with VDZ.

Recent benchmarking studies in FFPE are broadly consistent with this study (13, 15–18, 27). Collectively, these studies highlight that performance differs across platforms and applications and that panel design, segmentation, and tissue context remain major determinants of data quality and biological resolution. In that context, our study adds a clinically relevant benchmark in archived mucosal biopsies from inflammatory disease, while also showing that the main value of this optimization is biological, namely, improved resolution of colitis-associated cellular networks and treatment-response signatures. It is important to highlight that our customized panel for IBD, which included key landmark genes for our cell subsets of interest and additional genes to capture treatment responsiveness, performed better than larger panels meant for universal application. Because the probes per gene target are constrained by optical crowding and inversely relate to panel size, the focused custom panel improved our ability to resolve transcriptionally distinct cell subsets. Although it would be appealing to employ an unbiased universal panel across tissues, our results suggest that an optimized panel for a specific tissue type and disease state will capture more cells and genes of interest with improved performance. In IBD, there are ample reference tissue scRNA-seq datasets to guide panel design (1–4, 6, 7, 9), but other tissues and disease processes may benefit from initial studies using larger, unbiased panels or whole-transcriptome approaches. Multiple complementary spatial transcriptomic approaches might be necessary to adequately capture the transcriptional diversity of cell subsets and disease states in FFPE tissue.

The identification of both B cell abundance in GALT and IAF-monocyte networks in UC biopsies, including some VDZ nonresponders, complements recent studies regarding UC and the effects of VDZ (9, 25). Comprehensive flow cytometry analysis of mucosal biopsies identified a significant reduction of CD1c^+^ (BDCA1^+^) type 2 conventional dendritic cells (cDC2s) in UC patients on VDZ (28). We subsequently reported a shift of α4β7^+^ cDCs from the tissue to circulation in UC patients on VDZ (9). Interestingly, another group reported that VDZ reduced naive B and T cells and gut-homing plasmablasts, culminating in GALT attrition in VDZ responders (25). Given the central role of cDCs in priming naive T and B cells in lymphoid tissue, VDZ may reduce GALT by inhibiting migratory cDCs from trafficking to the colon. Here, we identified higher B cell abundance and *CD1C* expression in some VDZ pretreatment nonresponders by Xenium. Given that both B cells and CD1c^+^ cDCs localize to GALT, these data imply that a higher pretreatment GALT area may be associated with VDZ nonresponse, although the GALT signature in VDZ nonresponders was not validated in an external transcriptomic dataset. Recruitment of neutrophils and monocytes to inflamed colon tissue by IAFs has also been associated with UC and nonresponse to VDZ (8, 9). In this study, we additionally observed an increased abundance and trend toward proximity of IAFs and monocytes in UC compared with HC, confirming prior associations of this cellular network with colitis. Neutrophils were also increased in abundance in patients with UC in this study, and given similarities in gene expression, there is likely some overlap in the annotation of neutrophils and monocytes using spatial transcriptomics. Interestingly, there may be 2 subtypes of nonresponders: those with spatial profiles dominated by an innate inflammatory infiltrate and those with expanded adaptive immune subsets. This interpretation was further supported by unbiased CellCharter analysis, which identified an epithelial neighborhood enriched in responders and 2 distinct nonresponder neighborhood archetypes corresponding to innate IAF-monocyte-neutrophil and adaptive GALT-associated states. Although additional patients will be needed to confirm and further characterize tissue heterogeneity among UC treatment nonresponders, these findings suggest the presence of at least 2 distinct mechanisms of resistance to VDZ. Finally, we identified pretreatment spatial transcriptomic signatures in the epithelial compartment associated with a response to VDZ, suggesting a potential mechanism for mucosal healing. Namely, VDZ responders may have IECs that are poised to regenerate the epithelium once inflammation is suppressed.

There are important limitations of this study to consider and several caveats regarding spatial transcriptomics in general. First, this study is primarily descriptive and hypothesis-generating. Although spatial colocalization and neighborhood analyses identified reproducible disease- and treatment-associated cellular networks, further studies are needed to establish direct biological interaction and mechanism. Functional perturbation of these networks, particularly the IAF-myeloid and GALT-associated programs identified here, is an important next step. We also compared healthy and inflamed colon biopsies and VDZ responders versus nonresponders using a small number of patients. Even though the cohort size here is comparable with prior VDZ transcriptomic studies, the VDZ response analyses will require validation in larger multisite cohorts. Future retrospective case-control spatial transcriptomic studies also need to include additional therapies to identify signatures predictive of response or nonresponse across different therapies. We also focused exclusively on archived human FFPE colon tissue, which is a unique tissue type. These endoscopic mucosal biopsies are placed directly in formalin within seconds of collection, they permeabilize easily due to their small size, and embedding is typically completed within a day or a few days. Thus, our findings may not extrapolate seamlessly to other specimens that vary in size and fixation times, including surgical resection specimens. As noted above, several studies have examined imaging-based spatial platforms, and the results largely align with our study, but certain tissues or disease processes may perform differently in various platforms. It is important to acknowledge that spatial transcriptomic studies are also limited by sampling variability. For example, here we analyzed 4-mm-thick sections of cores from 2–3 mm biopsies meant to reflect the inflammatory status of an organ approximately 1 meter long. Adequate tissue sampling is critical to consider in all future spatial transcriptomic studies. Furthermore, block age and sample quality may play a more significant role when analyzing blocks older than 8 years. Lastly, inclusion and exclusion criteria are critical when using retrospectively identified, archived, clinical FFPE samples collected as part of standard of care rather than for a clinical trial.

In summary, we defined spatial immune-stromal niches in colitis and HCs using a custom, optimized, spatial transcriptomic workflow tailored for archived FFPE colon mucosal biopsies. This integrative approach enabled the identification of transcriptionally and spatially distinct fibroblast subsets and revealed a pronounced enrichment of IAF-myeloid cell neighborhoods in inflamed UC biopsies. This study adds to the growing body of literature identifying critical interactions among mononuclear phagocyte, neutrophil, and fibroblast subsets in inflamed tissues across multiple diseases. We also identified IAF-myeloid and GALT-associated archetypes of VDZ nonresponse together with epithelium-associated programs in responders, highlighting biologically distinct tissue states linked to treatment outcome. The capability of generating spatial transcriptomic data from routinely collected, archived clinical specimens with subcellular resolution could enable transformative translational research. Spatial transcriptomics will accelerate the identification and validation of candidate biomarkers and nominate novel therapeutic targets in affected tissue for various inflammatory disorders.

## Methods

### Sex as a biological variable.

Our study examined male and female human samples, but sex was not considered as a biological variable.

### Study participants and biospecimen collection.

For this study, archived FFPE samples were used. HC patients refer to those without known or suspected IBD who were undergoing routine colonoscopy or sigmoidoscopy for various indications, such as colorectal cancer screening. All specimens were processed similarly. Biopsies were placed in 10% formalin for up to 24 h, then ethanol, and then embedded in paraffin for long-term storage. Baseline demographic and clinical information about the study participants from the 3 datasets are provided in Supplemental Tables 1–3. The CosMx data from Dataset 1 were previously published and reanalyzed for this study (9). Information from Xenium and MERSCOPE analyses on Dataset 1 and Xenium analysis on Datasets 2 and 3 was acquired for this study as detailed below. We have consent to publish deidentified patient demographics, including age at the time of sample collection, sex, diagnosis, and medical center. Demographic options were defined by the investigators, and participants chose their classifications. Biopsy samples were categorized based on the level of inflammation observed endoscopically: noninflamed (score = 0), mildly inflamed (score = 1), moderately inflamed (score = 2), or severely inflamed (score = 3). Samples were assigned unique identifiers before further processing. Pretreatment Mayo endoscopic subscores were 2–3 for all UC patients. Response was defined as endoscopic remission (Mayo endoscopic subscore of 0–1) after completing induction, while nonresponse was defined as persistent moderate-to-severe inflammation (Mayo endoscopic subscore 2–3) after completing induction. The only exception was HS44, who had a pretreatment endoscopic subscore of 1 after failing 5-ASA therapy and a posttreatment score of 0. This patient was still classified as moderate-to-severe and higher risk colitis before VDZ given failure of first-line 5-ASA therapy but clearly responded to VDZ and achieved durable, deep histoendoscopic, steroid-free remission on VDZ. Biopsies from UC patients from Datasets 1 and 3 before treatment were assessed by a pathologist using the weighted Robarts Histopathology Index (RHI) (29, 30), blinded to endoscopic and clinical activity scores and other analyses. Quiescent disease was defined as RHI 0–3, mild disease as RHI 4–9, and moderate-severe disease as RHI ≥ 9 (31, 32).

### Technical performance comparisons between Xenium and CosMx spatial transcriptomic platforms, Xenium panel size, and Xenium segmentation strategy.

Our technical QC comparisons utilized transcript data, filtered to only include high-quality transcripts that passed platform-specific filters (Xenium platform Phred-scaled quality value (Q-score) > 20; all CosMx QC flags = pass) and our cell-level filtering schema, described in *Data preprocessing and annotation*. The sources of the data values used for various QC comparisons are detailed below. To quantify cellular location classification for transcripts, we used the Xenium transcript metadata Overlaps Nucleus metric (yes/no options) and the CosMx transcript metadata Cellular Compartment metric (0, cytoplasm, membrane, and nuclear options). After filtering, we had 0 transcripts with 0 values. For the cellular location classification plot, we grouped cells as nuclear and nonnuclear for both platforms. Cell area was compared across platforms using μm^2^ values. For Xenium, the cell area values were already provided in μm^2^. However, for CosMx, the cell area values were initially in pixels and were converted to μm^2^ by multiplying each pixel value by 0.0144, as 1 pixel equals 0.12 μm. Negative probes per cell were quantified using the Control Probe Counts cell metadata column in Xenium and nCount NegProbes cell metadata column in CosMx. Signal over background metrics were calculated by dividing the mean expression of each gene within cells by the mean negative probe counts per cell.

### Data preprocessing and annotation.

We developed and optimized a standard preprocessing pipeline using the Python packages scanpy, squidpy, and anndata, tailored for use with CosMx, Xenium, and MERSCOPE data (33, 34). We created spatial data objects in Python for each data type using cell-level gene expression, metadata, and spatial location information. 10x Genomics’ Xenium Analyzer software automatically removes low-quality transcripts (as defined by a Q-score < 20) from the cell feature matrix and secondary analyses. We included several orientation cores on the Xenium slides for Datasets 2 and 3 for TMA identification purposes. We removed the cells associated with these cores from our spatial data objects before proceeding with preprocessing. For the CosMx data and Xenium Datasets 1 and 2, we filtered out cells with <50 counts and <10 genes and genes with <1 count and detected in <10 cells. We modified our filtering schema for Xenium Dataset 3: removing cells with <30 counts instead of <50 and adding a nucleus filter (removing cells with 0 nuclei and >1 nuclei, unless the multinucleated cells expressed at least 2 out of 4 key neutrophil genes: *S100A8*, *S100A9*, *CSF3R*, *FCGR3B*). As the MERSCOPE dataset had very few cells passing QC, we modified our filtering schema to remove cells with <10 counts instead of <50. After filtering, we proceeded to normalize and log-transform the data and regress out unwanted sources of variation, specifically the number of transcripts per cell and the number of unique (gene) transcripts per cell. Next, we scaled the data and ran a principal component analysis. We computed a neighborhood graph using n_neighbors = 10 and n_pcs = 30 (CosMx, Xenium) or n_pcs = 10 (MERSCOPE). We embedded the neighborhood graph using UMAP, specifying min_dist = 0.2 and spread = 1.5 (CosMx, Xenium) or spread = 1.75 (Xenium, MERSCOPE), and clustered using Leiden community detection. All clustering used resolution = 1, except for Xenium Dataset 2 5k and Dataset 3, which used resolutions of 1.6 and 1.3, respectively. We annotated cell clusters by known markers and spatial location using the CZ CELLxGENE tool for refinement (35, 36). The CosMx and Xenium datasets were processed with the same pipeline and settings, as noted above.

### Integrating Xenium replicates and batches.

For Xenium Dataset 1 and Dataset 2 290-plex, we generated a replicate run from separate sections of the same TMA, and for Dataset 3, we processed 2 slides containing unique samples that served as batches. These replicates and batches were integrated for most downstream analyses. We created spatial data objects in Python for each Xenium slide using cell-level gene expression, metadata, and spatial location information. We added prefixes to the cell ID values to distinguish between slides 1 and 2 and added a “replicate,” “batch,” or “slide” metadata column. We proceeded to filter the datasets separately with the filtering schema noted above. Next, we concatenated the 2 anndata objects along the observations axis (*x* axis; rows) using the anndata.concat function. This process preserves all subelements of each object, while stacking the observations in an ordered manner. By concatenating along the observations axis, we effectively combined data from different cells (observations) into a single dataset. We then added 15,000 to the x-coordinate value of each cell in slide 2 to offset its value from those in slide 1. This adjustment allowed us to visualize the spatial coordinates of the concatenated dataset with slides 1 and 2 displayed side by side rather than overlapping. After concatenating, we proceeded with the preprocessing pipeline as usual.

### Neighborhood enrichment.

We performed neighborhood enrichment analyses for fine annotation cell clusters within the Xenium HC and UC PRE data subsets for Dataset 1 and Xenium ICI for Dataset 3 using functions from the squidpy package. To do this, we constructed a spatial nearest-neighbor graph using Delaunay triangulation, which links cells based on their spatial proximity to each other within a connectivity matrix. We then calculated an enrichment *z* score for each pair of fine annotation cell clusters based on cell proximity within the connectivity graph. These analyses were conducted for the Xenium HC and UC PRE data subsets in 2 ways: (a) by calculating neighborhood enrichment *z* scores for each pair of fine annotation cell clusters across the entire dataset (all cores, all patients) and (b) by calculating neighborhood enrichment *z* scores specifically for our cell types of interest, Fibroblast_IAF and MNP_monocyte cell clusters, within each individual core and then grouping these *z* score values for each unique patient. Exploratory Xenium ICI analysis was performed by calculating neighborhood enrichment *z* scores for each pair of fine annotation cell clusters across the entire dataset (all cores, all patients).

### Unbiased CellCharter analysis on combined Xenium datasets.

CellCharter (26) was used to analyze Datasets 1 and 3, jointly reprocessed as a combined object and filtered on the shared 275 genes. We integrated the data using Harmony at the panel level rather than by slide. A spatial neighbor graph was constructed using squidpy’s Delaunay triangulation, followed by pruning of long-range connections using CellCharter’s remove_long_links function. We then applied CellCharter’s autok procedure to determine the optimal number of clusters (k) based on cluster stability, while also prioritizing a relatively fine resolution to capture heterogeneous biological features among UC PRE NR patients. Based on these criteria, we selected k = 8. Resulting clusters were subsequently merged as needed and annotated. Several CellCharter clusters corresponded closely with our UC PRE R and UC PRE NR gene signatures. CellCharter cluster annotations were informed by examining enrichment of annotated cell types within each cluster. To further evaluate this correspondence, we calculated per-cell gene signature scores based on the mean expression of genes within each signature for Datasets 1 and 3. For each signature, cells were ranked by their signature score, and the top 25% were classified as high-expressing. We then quantified the proportion of these high-expressing cells assigned to each CellCharter cluster. CellCharter clusters corresponding to a given gene signature were enriched for both the expected cell types and cells with high expression of that signature, supporting concordance between spatial neighborhoods and gene signature activity.

### Pseudobulk DEG analysis and GSEA.

Spatial transcriptomic data were used to perform pseudobulk DEG analysis using DESeq2 (37). Three distinct pseudobulk DEG analyses were conducted on Dataset 1: CosMx data comparing UC PRE with HC, Xenium data comparing UC PRE with HC, and Xenium data comparing UC PRE nonresponders with UC PRE responders. Two distinct pseudobulk DEG analyses were performed on Dataset 3, comparing ICI with UC PRE and comparing ICI with HC. Samples were stratified by patients. The bulk transcriptomic dataset (GSE73661) (21), consisting of colonic biopsies from HC and UC patients before and after various biologic treatments, was obtained from the Gene Expression Omnibus (GEO) database and utilized for GSEA. For this analysis, the bulk transcriptomic samples were categorized into HC (*n* = 12) and UC PRE (*n* = 43), or UC PRE VDZ R (*n* = 11) and UC PRE VDZ NR (*n* = 9). Data were normalized and *z* scored before being processed in the GSEA program (version 4.4.0) (38). HC and UC PRE signatures were defined based on the pseudobulk DEG analysis from Xenium spatial transcriptomic data. For CosMx, only the UC PRE gene signature was identified. The number of permutations was set to 1,000, with no dataset collapse, using the Affymetrix Human Gene 1.0 ST Array and *t* test. For each analysis, a NES was calculated, and only NES values with a *P* value < 0.05 and an adjusted *q* value (FDR) < 0.1 were considered significant. Gene signatures identified by Xenium for UC PRE R (IEC_R) and UC PRE NR (IAF-monocyte-neutrophil_NR and GALT-B-DC-S4_fibroblast_NR) were further explored in Datasets 1 and 3, as well as in an external cohort of patients before VDZ treatment, stratified as UC PRE R and UC PRE NR. These GSEAs were performed using the fgsea package in R v1.35.6, which implements an algorithm for fast GSEA (39), and leading-edge genes were defined in each dataset as the genes that drive the enrichment in each specific signature. Detailed gene signatures are reported in Supplemental Table 11.

### Statistics.

For comparative analyses across spatial transcriptomic platforms, differences were assessed using a nonparametric, 2-sided Mann-Whitney test. For Dataset 3, plots examining the number of transcripts and unique features per cell per block were analyzed using Spearman’s correlation, with the correlation coefficient (ρ), *P* value, and slope calculated to assess differences in population medians across FFPE blocks ranging from 1 to 11 years of age. Block age was measured from the time of tissue collection until the assay was conducted. Cell frequencies for Xenium Dataset 1 comparing 2 groups were also assessed using the Mann-Whitney test, followed by the 2-stage linear step-up procedure of Benjamini, Krieger, and Yekutieli, which adjusts for multiple comparisons by controlling the FDR. Cell frequencies for Xenium Dataset 3 comparing 3 groups were assessed using a Kruskal-Wallis test with FDR correction. The *q* value, representing the FDR-adjusted *P* value, was set for discovery at *q* < 0.1. For DeSeq2 analysis, significance thresholds were defined as log_2_FC > 0.4 or < –0.4 and *q* < 0.1 and count threshold of baseMean > 500 for Xenium and > 400 for CosMx (Supplemental Tables 6–10). Additional statistical analyses were performed using GraphPad Prism 10. The ComplexHeatmap R package was used to generate expression *z* score heatmaps for DEGs.

### Study approval.

The study was conducted according to the Declaration of Helsinki principles and was approved by the Institutional Review Board of UCSF (19-27302). Written informed consent was received from participants prior to inclusion in the study. For retrospective retrieval, study subjects were identified by querying the electronic medical records of patients previously seen by UCSF Gastroenterology with existing archived specimens. This was followed by obtaining written informed consent and approval.

### Data availability.

Raw and annotated anndata objects are available on FigShare for the following datasets: Xenium, https://doi.org/10.6084/m9.figshare.27327813 and https://doi.org/10.6084/m9.figshare.31914375; MERSCOPE, https://doi.org/10.6084/m9.figshare.27327828; and CosMx reanalysis, https://doi.org/10.6084/m9.figshare.27327861 For datasets integrated for downstream analyses, we have included the combined anndata objects. Individual slides can be viewed by subsetting on the “batch” or “slide” metadata columns. Raw Xenium and MERSCOPE data files are available in the GEO under accession numbers GSE282123 and GSE282124, respectively. The original CosMx data used in this study were previously published (GSE250498, phs003502.v1.p1, and https://doi.org/10.6084/m9.figshare.23896959.v3) (9). Publicly available microarray data (GSE73661) (21) were downloaded from GEO. The numerical values underlying all graphs in the figures are available in the Supporting Data Values file.

All code necessary for recreating the reported analyses and figures is available on the single-cell spatial transcriptomics of FFPE colon biopsies project GitHub repository (https://github.com/UCSF-DSCOLAB/spatial_transcriptomics_colitis_analysis_2024; commit ID ef610b3). This includes R markdowns, Jupyter notebooks, and Conda environment YAML files.

## Author contributions

Conceptualization: GKF, MGK, and AJC. Patient recruitment and sample collection: GL, JHH, UM, DYO, and MGK. Sample retrieval and histology: EM, GL, JHH, MGK, CJB, and RMG. Sample processing and data acquisition: EM, VJ, CAE, GL, JT, JHH, and WE. Cell subset annotation: EM, GL, DEL, JYH, and MGK. Spatial transcriptomic analysis: EM and MLL. Supervision: GKF, MGK, and AJC. Funding acquisition: GKF, MGK, and AJC. All authors contributed to manuscript preparation. EM and MLL contributed equally, and their order was assigned by chronology of effort. GL, JHH, and VJ contributed equally, and their order was assigned by chronology of effort. GKF, MGK, AJC contributed equally, and their order was determined by mutual agreement.

## Conflict of interest

The Kattah, Combes, and Fragiadakis labs receive research support from Eli Lilly. The Combes lab receives research support from Genentech. MGK has consulted for Sonoma Biotherapeutics, Santa Ana Bio, Spyre, Cellarity, Vedanta, and CytoMx, and he serves on the scientific advisory board for Switchback Therapeutics. AJC is a member of the scientific advisory board of Foundery Innovations and has received consulting fees from Survey Genomics. UM is a consultant for Abbvie, Janssen, Takeda, Pfizer, BMS, Gilead, Enveda, Lilly, Merck, Rani Therapeutics, Celltrion, and Abivax and received grant support from Leona and Harry Helmsley Charitable Trust. CJB has consulted for CytoMx. DYO has consulted for Revelation Partners and has received research support from Merck, PACT Pharma, the Parker Institute for Cancer Immunotherapy, Poseida Therapeutics, TCR2 Therapeutics, Roche/Genentech, and Nutcracker Therapeutics as well as travel and accommodations funds from Roche/Genentech and Poseida Therapeutics. The expanded 5k panel was provided at no cost by 10x Genomics, but all other costs were paid for using the funding described below.

## Funding support

This work is the result of NIH funding, in whole or in part, and is subject to the NIH Public Access Policy. Through acceptance of this federal funding, the NIH has been given a right to make the work publicly available in PubMed Central.

- NIH grants R01DK14167 (to MGK); U01DE028891-01A1, R01AI093615-11, R01DK103735, P30AR070155-05, U01AI168390, R01AI170239, P30AI027763-31, and R01DE032033 (to GKF); and R35CA24244, R01HL181372, R01HL175312, and R35HL171251 (to AJC).
- Benioff Center for Microbiome Medicine Faculty Project Award (7031446 to MGK).
- Career Award for Medical Scientists from the Burroughs Wellcome Fund (to MGK).
- Chan Zuckerberg Initiative (to GKF).
- Gates Foundation (to GKF).
- Eli Lily (to GKF).
- UCSF Bakar ImmunoX Initiative (to GKF and AJC).
- Melanoma Research Alliance (to AJC).
- UCSF Program for Breakthrough Biomedical Research (to AJC).
- The Leo Foundation (to AJC).
- The Cancer Research Institute (to AJC).
- UCSF ImmunoX.
- Kenneth Rainin Foundation.

## Supplementary Material

Supplemental data

Supplemental tables 1-11

Supporting data values

## Version 1 06/09/2026

In-Press Preview

## Version 2 07/15/2026

Electronic publication
