# Supplementary material for: Single-cell spatial transcriptomics of formalin-fixed, paraffin-embedded biopsies reveals colitis-associated cell networks
Source: J Clin Invest. 2026 Jun 9;136(14):e202488. doi: 10.1172/JCI202488 (PMC13367960; doi:10.1172/JCI202488)
Supplement: Supplemental data [file jci-136-202488-s338.pdf]

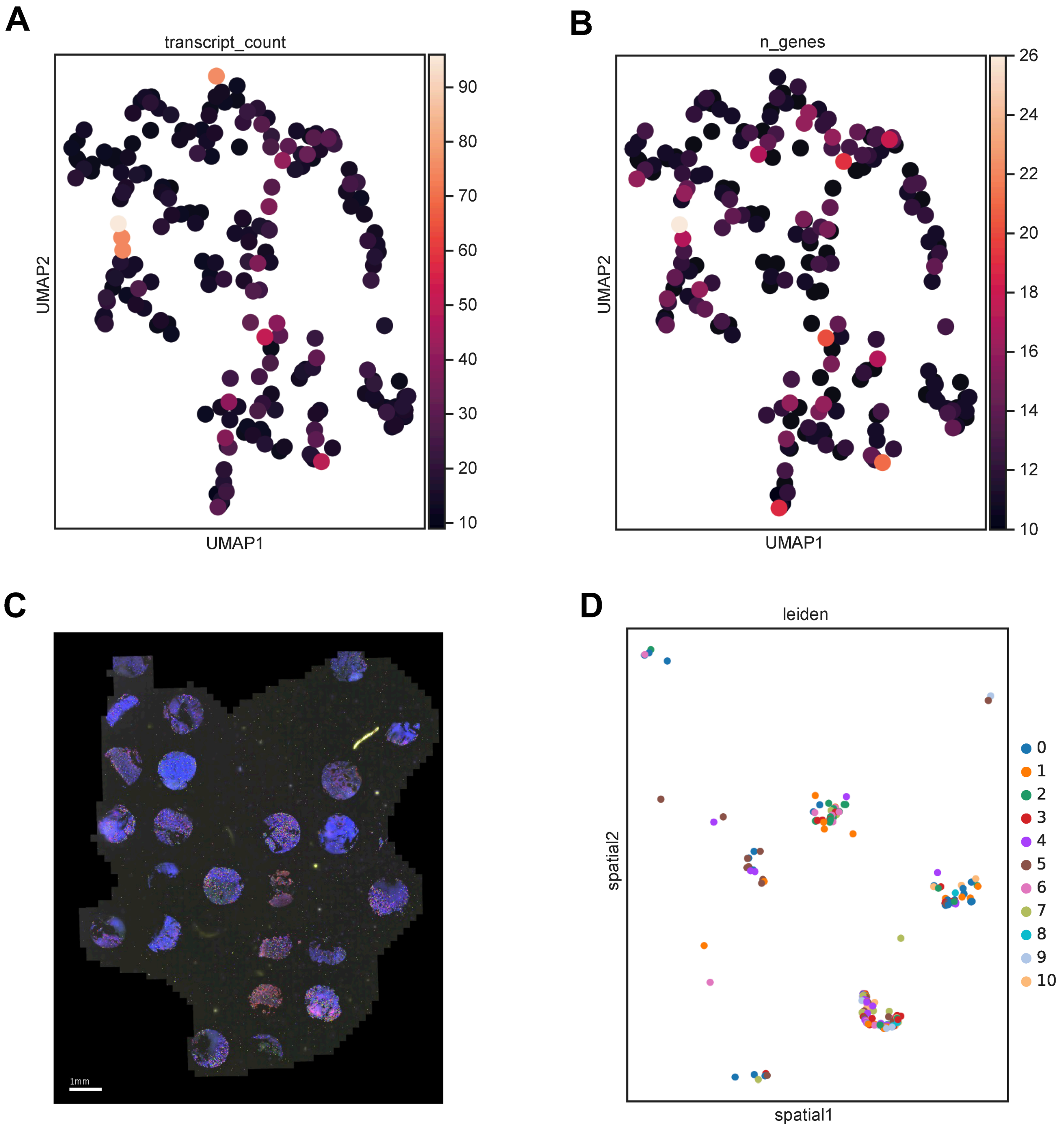

Supplemental Fig. 1. MERSCOPE analysis. (**A,B**) UMAP visualization of MERSCOPE in *Dataset 1* (212 cells), highlighting (**A**) transcript count per cell, (**B**) number of genes per cell, and (**C**) area of TMA scanned showing DAPI and detected transcripts. (**D**) Spatial scatter plot depicting the spatial location of cells in the MERSCOPE dataset in relation to the TMA slide, colored based on leiden clustering.

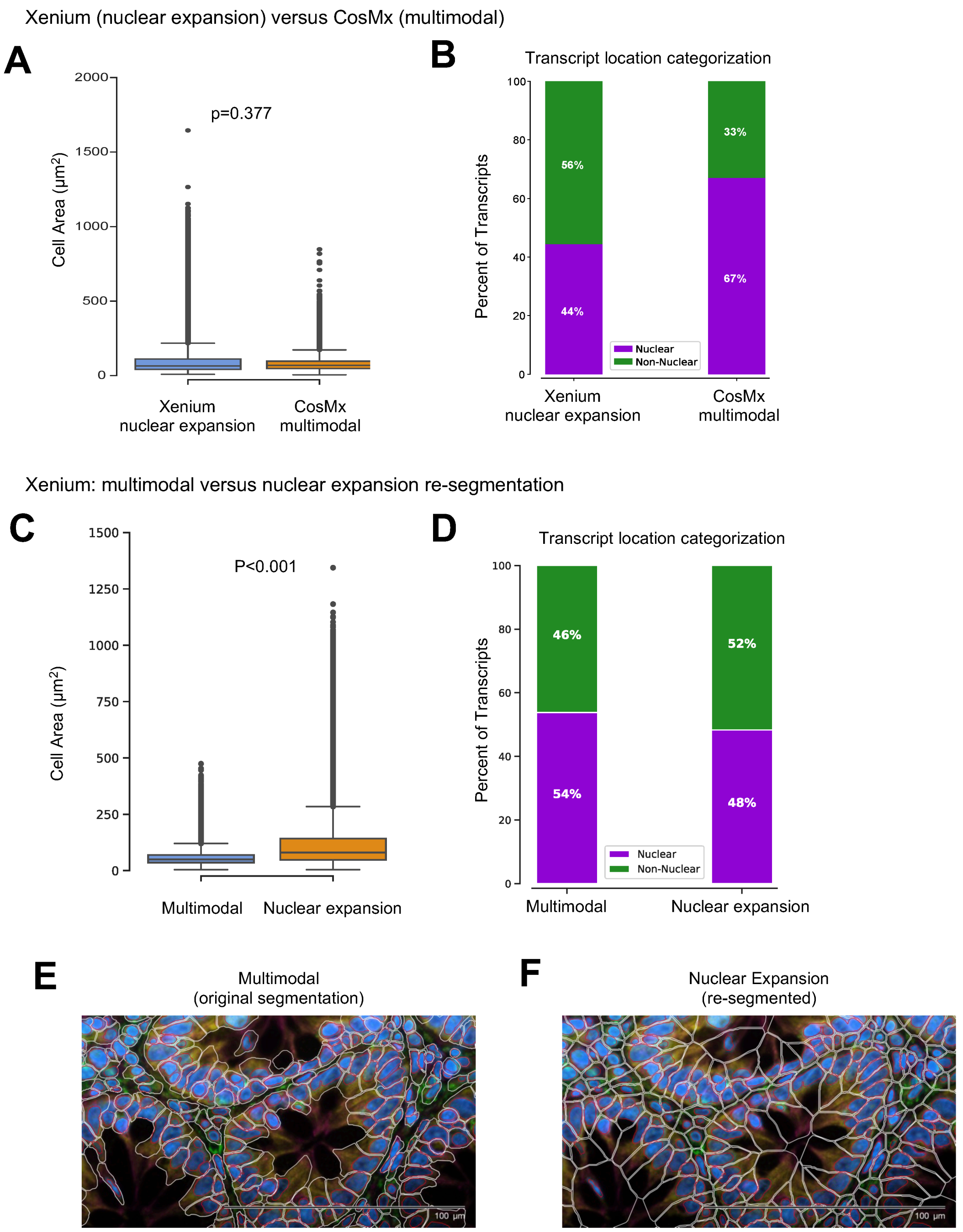

Supplemental Fig. 2. Segmentation comparison across and within iSCST platforms. (A,B) *Dataset 1* Xenium (nuclear expansion) versus CosMx (multimodal) comparison. **(A)** Cell area (µm^2) per cell in Xenium and CosMx datasets; data presented as median and interquartile range (IQR). **(B)** Stacked bar plot comparing the percentage of nuclear versus non-nuclear transcripts within each dataset. **(C-F)** *Dataset 2* Xenium 5k-plex comparing multimodal segmentation versus the same cells re-segmented using nuclear expansion, highlighting **(C)** cell area (µm^2) per cell, presented as median and IQR, **(D)** percentage of nuclear versus non-nuclear transcripts, and **(E, F)** representative cell segmentation visualizations. DAPI nuclei (blue), nuclei outlines (red), cell borders (white). P values for panels **A** and **C** were calculated using two-sided Mann-Whitney U tests.

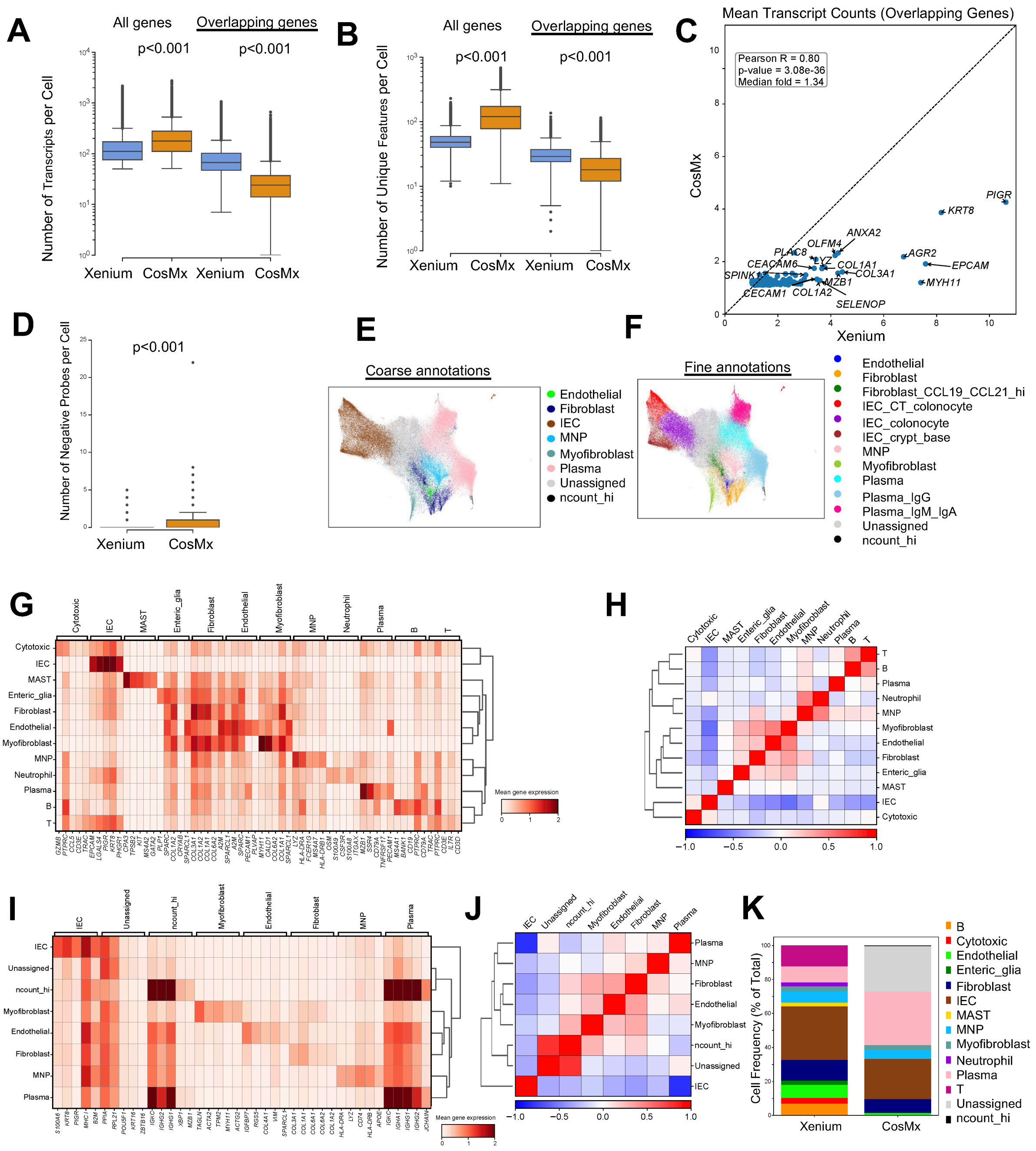

Supplemental Fig. 3. Xenium and CosMx dataset quality control and cell mapping-related metrics. (A,B) Number of **(A)** transcripts and **(B)** unique features detected per cell within Xenium and CosMx datasets, calculated using the complete gene panel for each platform (Xenium, 290 genes; CosMx, 1,000 genes; left) and limited to the 159 overlapping genes across both panels (right); data presented as median and IQR. Transcript counts and unique feature counts were compared between Xenium and CosMx using two-sided Mann-Whitney U tests. **(C)** Correlation between Xenium and CosMx mean transcript counts for the 159 overlapping genes was assessed using the Pearson correlation coefficient (two-sided test)**. (D)** Number of negative probes detected per cell in Xenium and CosMx (Xenium mean=0.03 and CosMx mean=0.37), presented as median and IQR and compared using a two-sided Mann-Whitney U test**. (E,F)** UMAP visualization of CosMx dataset (126,368 cells), colored by coarse **(E)** and fine **(F)** annotations. **(G-I)** Heatmap displaying gene expression of the top 5 landmark

genes for Xenium **(G)** and CosMx **(I)** coarse annotation, and correlation matrix displaying the correlation between Xenium **(H)** and **(J)** coarse annotation cell types. **(K)** Stacked bar plots displaying cell frequency (percent of total) for Xenium and CosMx coarse annotations.

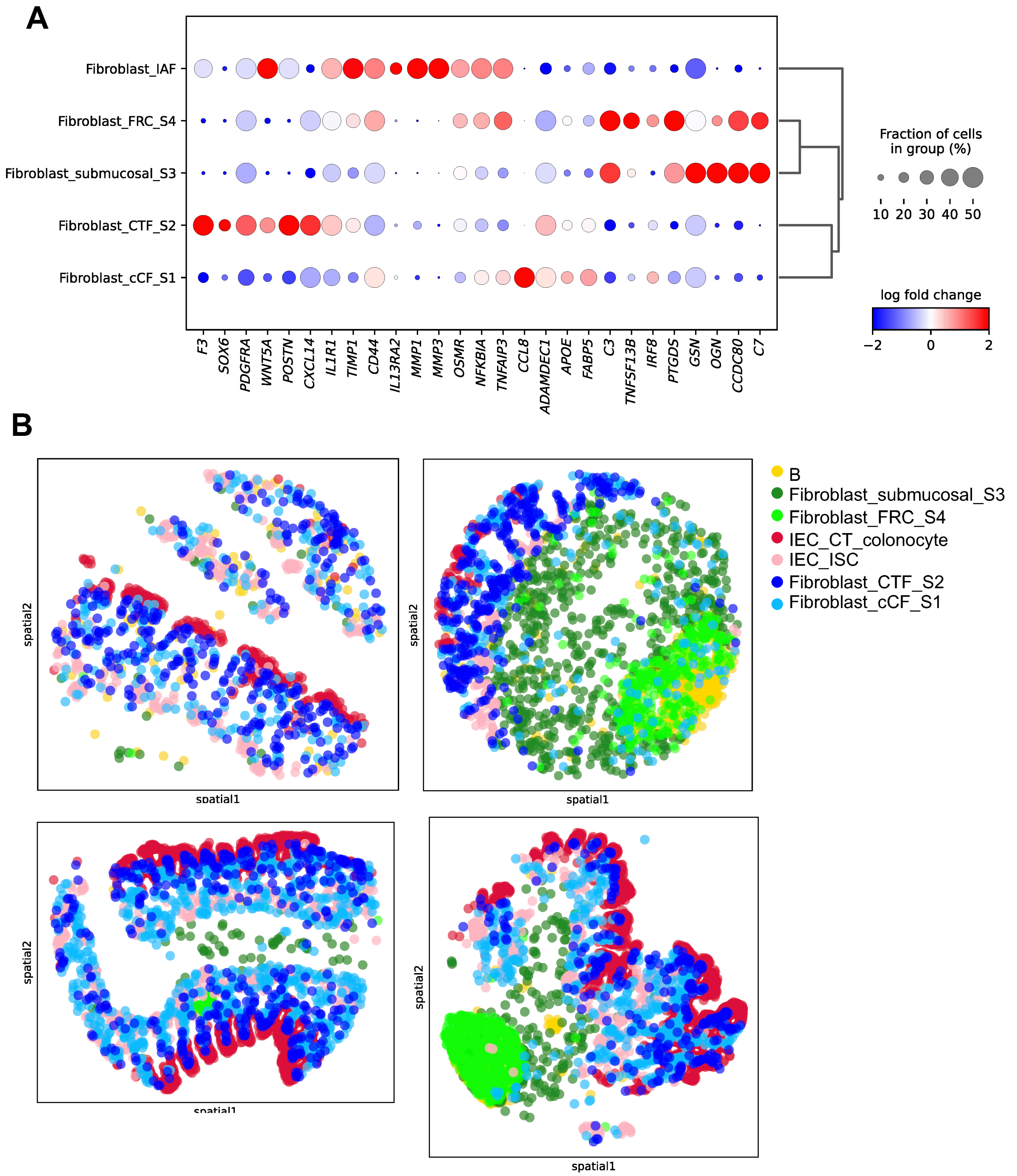

Supplemental Fig. 4. Xenium enabled identification and spatial localization of distinct fibroblast subsets in colon mucosal biopsies. (**A**) Dot plot representation of landmark genes for the indicated subsets. **(B)** Transcriptionally distinct fibroblast subsets identified by relative spatial localization in colon tissue from representative cores for the indicated cell subsets. IAF, inflammation-associated fibroblast; IEC, intestinal epithelial cell; ISC, intestinal stem cell; FRC, fibroblastic reticular cell; cCF, colonic crypt fibroblast; CTF, crypt top fibroblast.

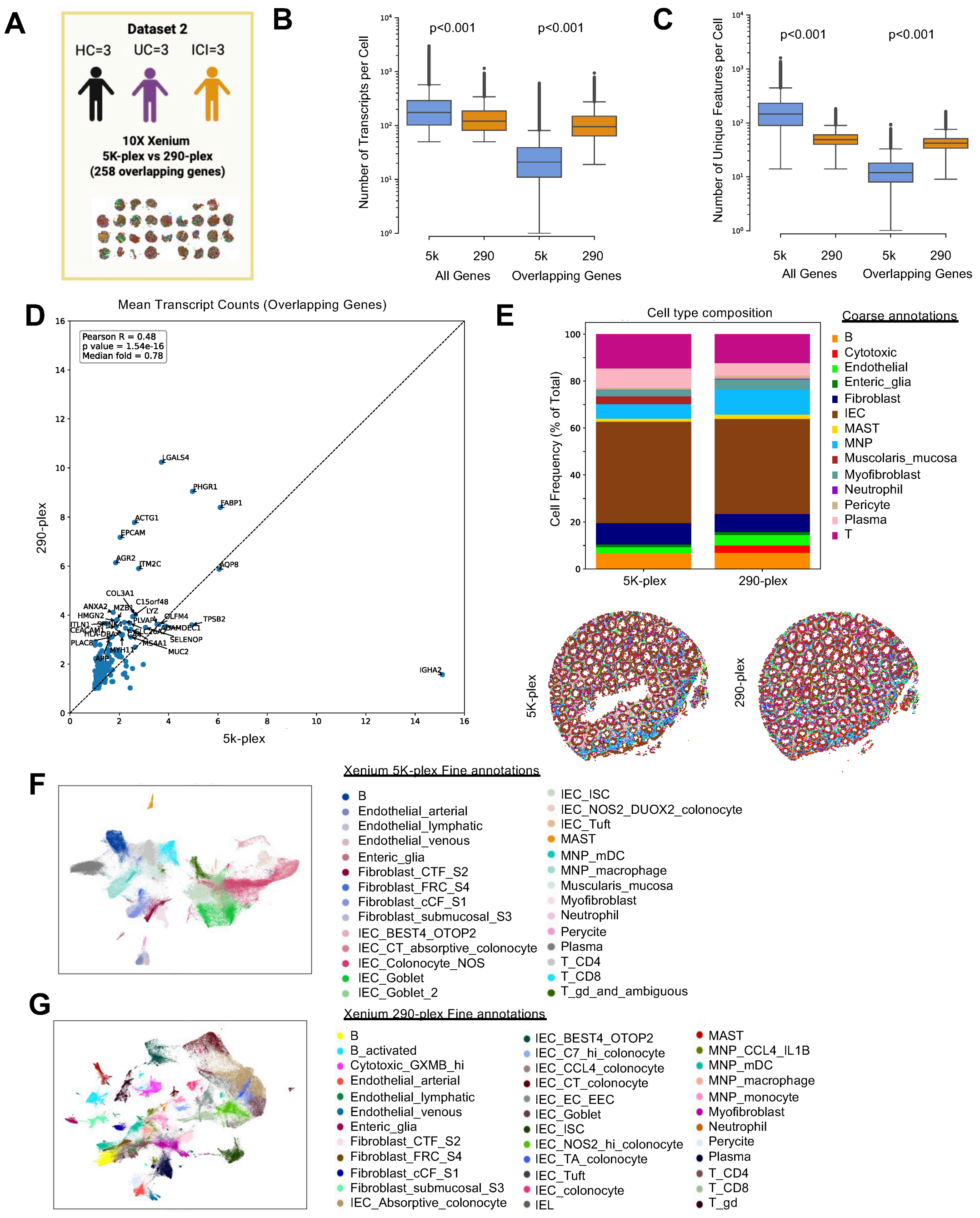

Supplemental Fig. 5. Comparative performance of custom 290-gene and 5k-gene panels on the Xenium platform. (**A**) Schematic for *Dataset 2.* Created with BioRender.com. (**B,C**) Number of (**B**) transcripts and (**C**) unique features detected per cell within the 290-plex and 5k datasets, calculated using the complete gene panel and limited to the 258 overlapping genes across both panels; data presented as median and IQR. Transcript counts and unique features were compared between the two Xenium panels using two-sided Mann-Whitney U tests. (**D**) Correlation of mean transcript counts for the 258 overlapping genes between the two Xenium panels was assessed using the Pearson correlation coefficient (two-sided test). **(E)** Stacked bar plots for coarse annotation displaying cell frequency (percent of total) for Xenium panels (top), representative scatter plots for matching cores from both Xenium panels coarsely annotated (bottom). (**F,G**) UMAP visualization colored by fine annotations for **(F)** 5k (260,611 cells) and **(G)** 290-plex (263,814 cells) datasets.

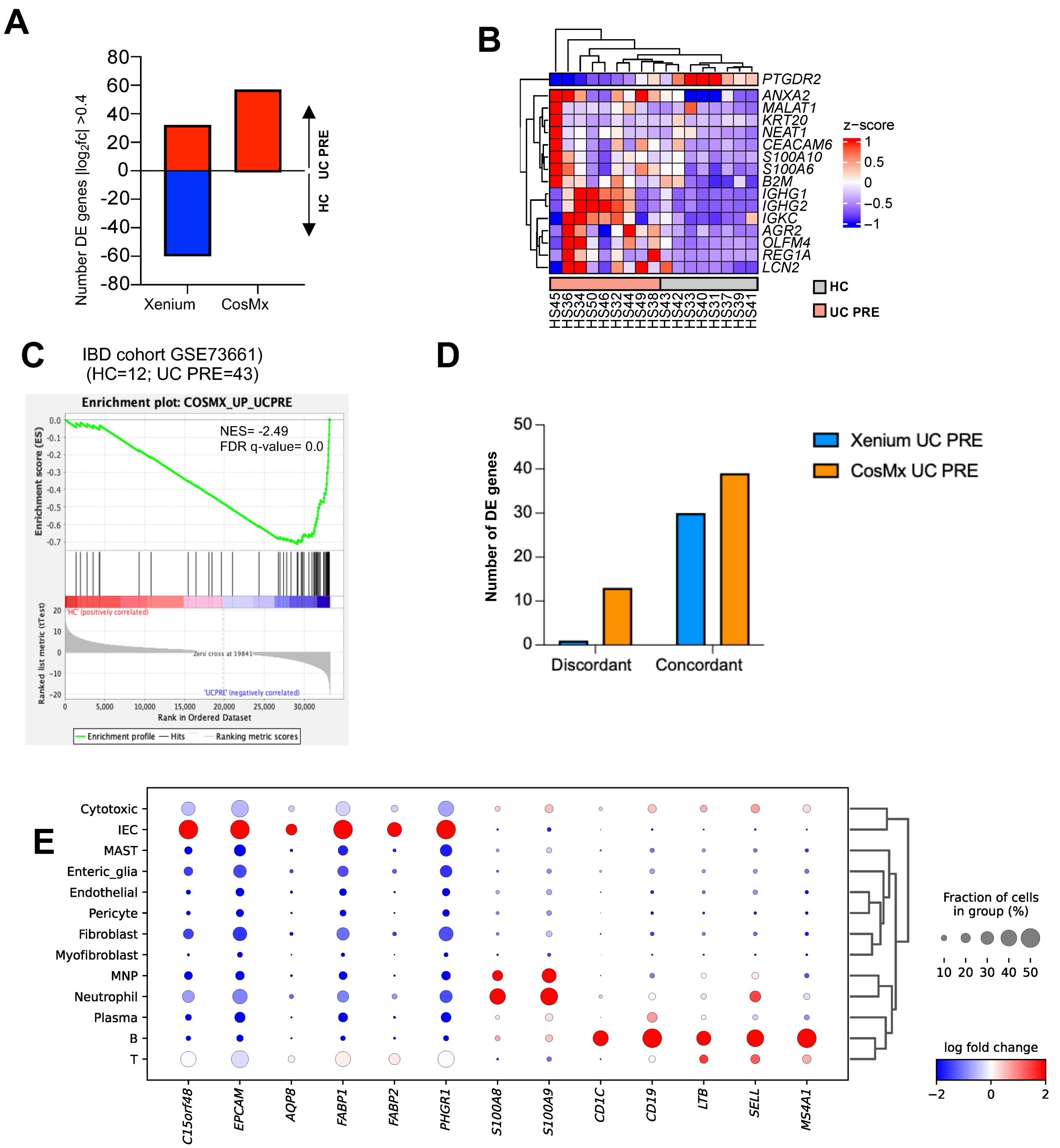

Supplemental Fig. 6. Platform comparison of transcriptomic signature detection and cell mapping of disease-associated features using iSCST. (**A**) Number of pseudobulk DE genes in the indicated platform with log2fc > 0.4 or <-0.4 in UC PRE relative to HC identified by DESeq2 analysis. (**B**) Heatmap of expression z-scores for the indicated genes in UC PRE (Up/Down) relative to HC for CosMx dataset. (**C**) GSEA of CosMx UC PRE spatial gene signature in an external cohort of patients and relative NES. (**D**) Number of DE genes in the UC PRE signature for Xenium and CosMx that are concordantly or discordantly expressed to the UC PRE patients from the publicly available dataset. (**E**) Dot plot representation of a subset of genes from pseudobulk DEG analysis by coarse annotations. For panel **B,** some genes are off-scale for visualization purposes, z-score set from -1 to 1.

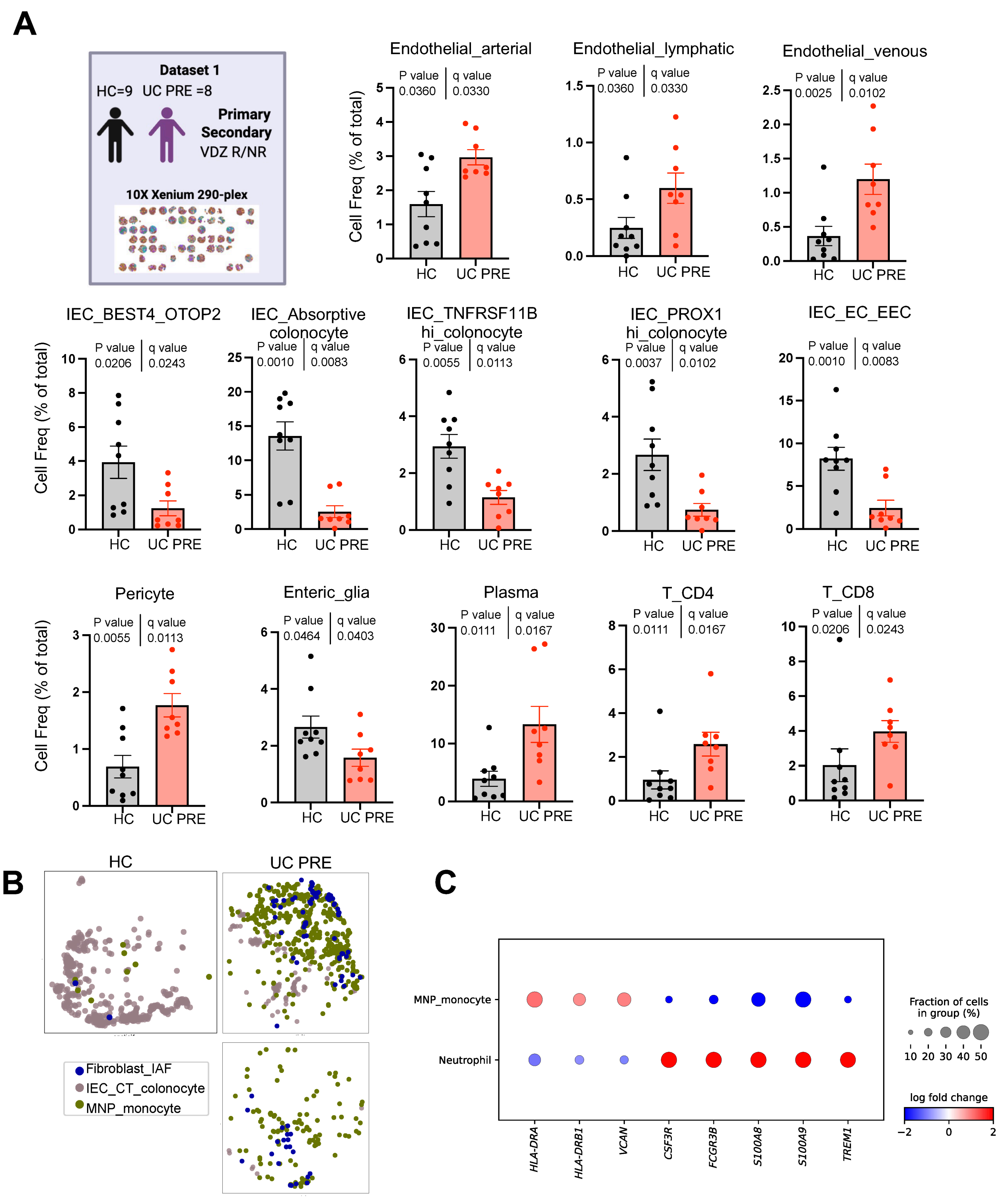

Supplemental Fig. 7. Cellular composition and spatial distribution differences in mucosal biopsies between healthy controls and ulcerative colitis patients. (**A**) Cell frequencies of the indicated subsets in *Dataset 1* comparing HC and UC PRE, each dot represents one patient (data presented as mean ± SEM); Mann–Whitney, two-sided test with FDR correction; p<0.05 and q<0.1 threshold for discovery. Only statistically significant cell subsets are shown with exact p-value and q-value displayed. **(B)** Spatial scatter plot of representative cores highlighting IAFs, crypt top (CT) colonocytes, and monocytes in HC and UC PRE biopsies. (**C**) Dot plot representation of landmark genes for the indicated subsets.

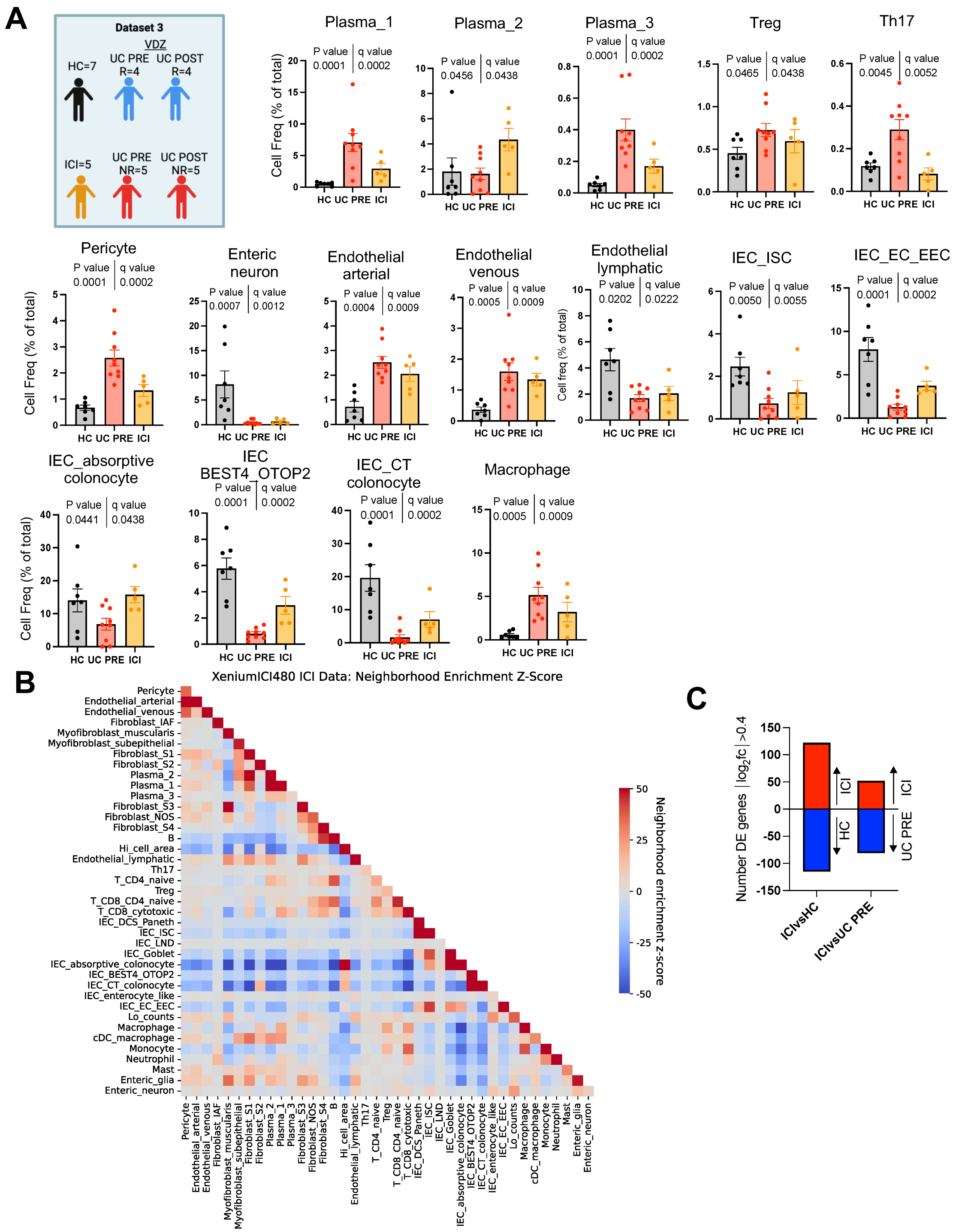

Supplemental Fig. 8. Cellular composition pseudobulk transcriptomic and neighborhood enrichment differences of mucosal biopsies between immune checkpoint inhibitor–induced colitis and ulcerative colitis. (**A**) Cell frequencies of the indicated subsets for *Dataset 3* (top left panel) comparing HC, UC and ICI, each dot represents one patient (data presented as mean ± SEM); Kruskal-Wallis, Dunn's multiple comparison test with FDR correction; p <0.05 and q<0.1 threshold for discovery. Only statistically significant cell subsets are shown with exact p-value and q-value displayed. (**B**) Heatmap displaying neighborhood enrichment z-scores for fine annotation cell pairs within ICI biopsy. (**C**) Number of pseudobulk DE genes in the indicated comparisons with log2fc > 0.4 or <-0.4 and q <0.1 were identified by DESeq2 analysis.

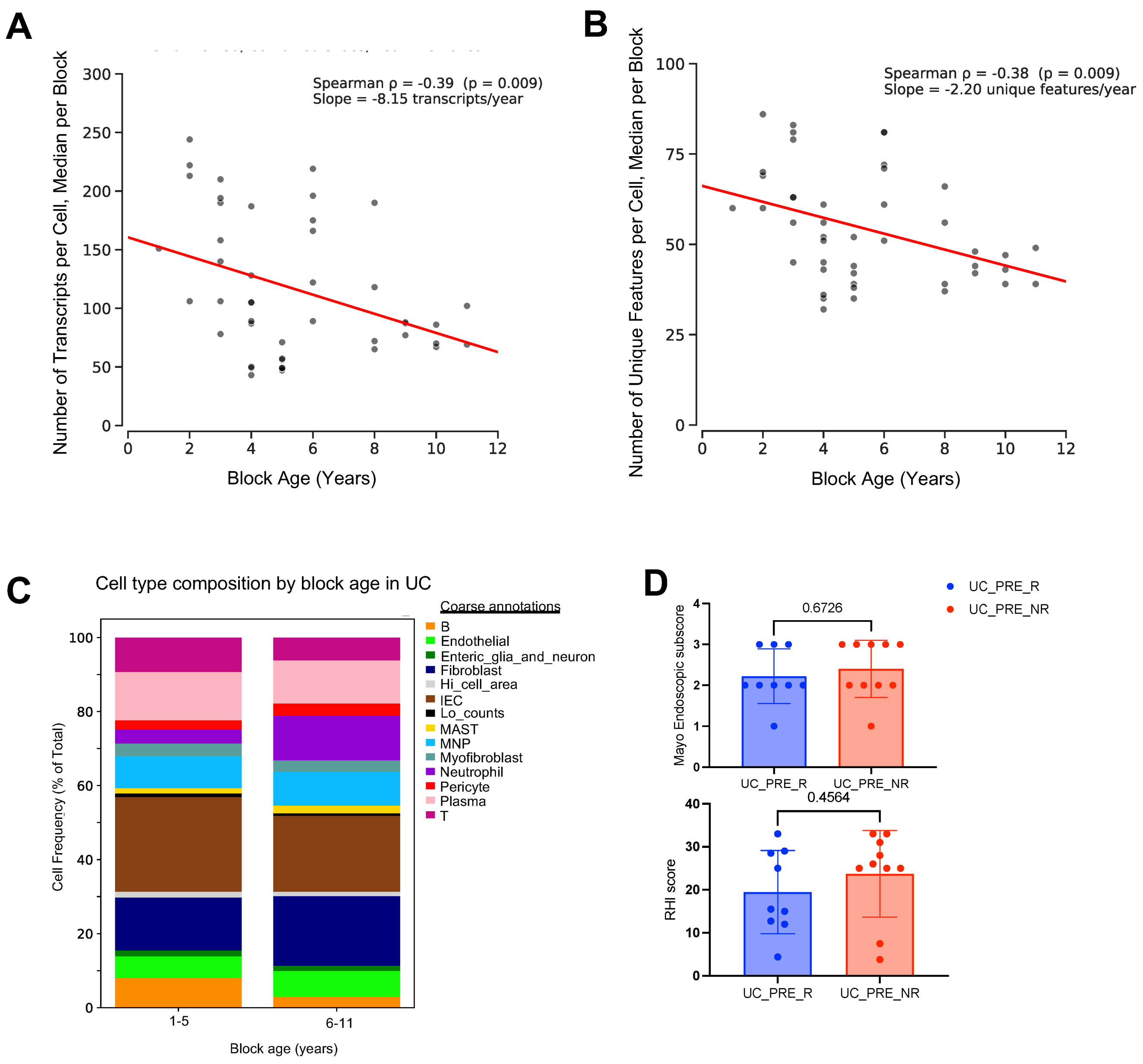

Supplemental Fig. 9. Xenium platform performance across tissue block ages and disease severity metrics for UC patients before treatment. Median number of (**A**) transcripts and (**B**) unique features detected per cell across tissue blocks of different ages. Associations with block age were assessed using Spearman's rank correlation coefficient; red lines indicate ordinary least squared linear fits. (**C**) Stacked bar plots for coarse annotation displaying cell frequency (percent of total) in UC tissue blocks, grouped by block age. (**D**) Bar plots showing the Mayo Endoscopic score per patient (top) and Robarts Histopathology Index (RHI) per patient (bottom), data presented as mean ± SD; Unpaired nonparametric Mann–Whitney, two-sided test.

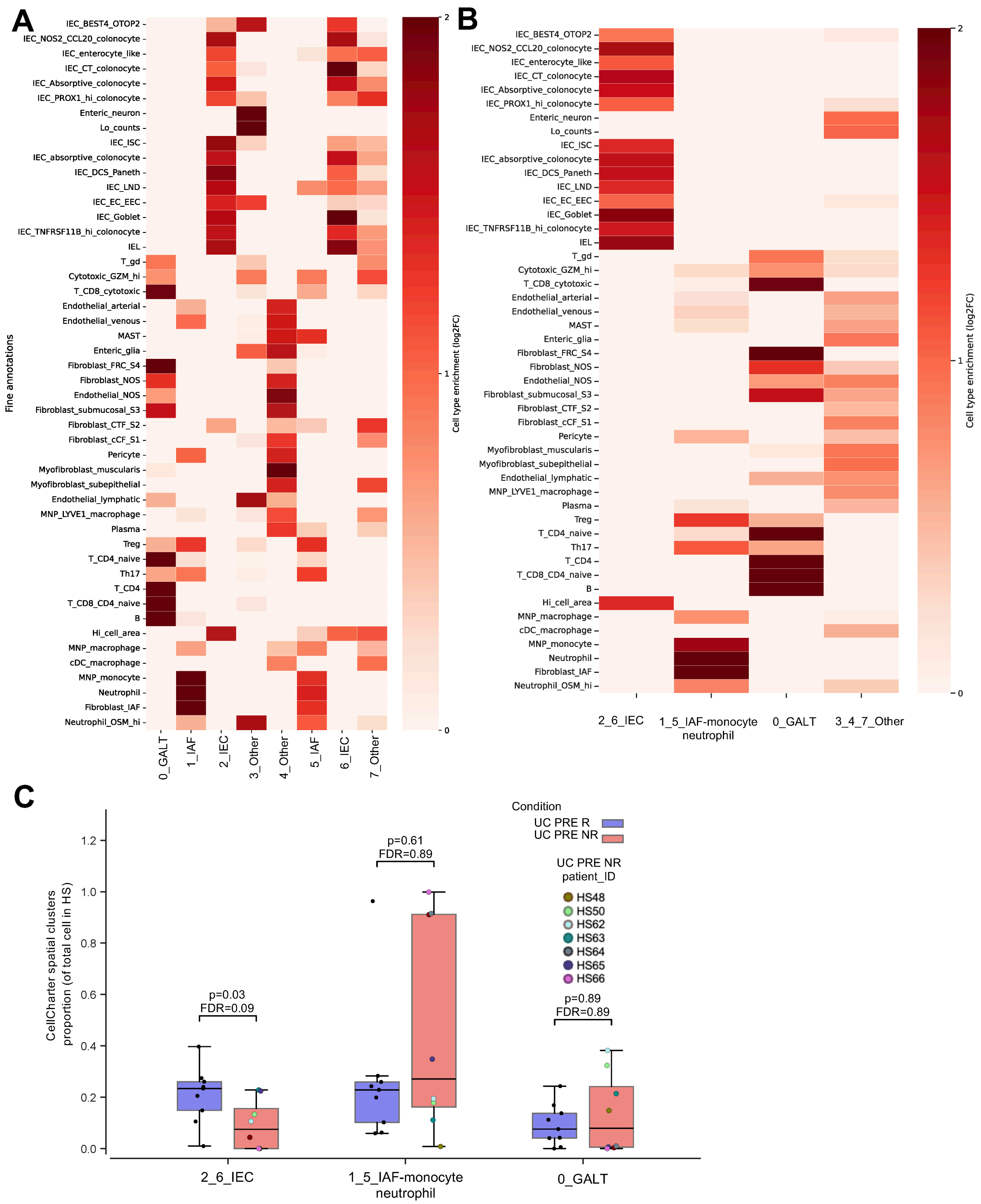

Supplemental Fig. 10. CellCharter analysis on *Datasets 1* and *3* identifies spatial clusters of response and non-response. (**A-B**) Heatmap showing the cell type enrichment score by (**A**) CellCharter spatial clusters (k=8) and by (**B**) merged-annotated CellCharter clusters. (**C**) CellCharter cluster proportions for the three selected response and non-response spatial clusters in UC PRE R and UC PRE NR patients, shown as a fraction of total cells per patient core. Each point represents an individual patient. Differences between groups were assessed using two-sided Mann-Whitney U tests with Benjamini-Hochberg correction applied across the three clusters. Raw and FDR-adjusted P values are shown.

SUPPLEMENTAL TABLE LEGENDS

Supplemental Table 1. Baseline demographic and clinical data for *Dataset 1*. Categorical variables were analyzed by Chi-square test and continuous variables were compared using one-way ANOVA with FDR correction or Mann-Whitney test where appropriate. ns, not significant; n/a, not applicable; pre, pre-VDZ treatment; post, post-VDZ treatment. VDZ, vedolizumab; y, years; mo, months.

Supplemental Table 2. Baseline demographic and clinical data for *Dataset 2*. n/a, not applicable; IFX, infliximab; ADA, adalimumab; ICI, immune checkpoint inhibitor induced-colitis; CPI, checkpoint inhibitor; y, years; w, weeks.

Supplemental Table 3. Baseline demographic and clinical data for validation *Dataset 3*. Categorical variables were analyzed by Chi-square test and continuous variables were compared using one-way ANOVA with FDR correction or Mann-Whitney test where appropriate. ns, not significant; n/a, not applicable; pre, pre-VDZ treatment; post, post-VDZ treatment. CPI, checkpoint inhibitor; ICI, immune checkpoint inhibitor induced-colitis; IFX, infliximab; VDZ, vedolizumab. y, years; mo, months; w, weeks.

Supplemental Table 4. Gene panels for the different spatial transcriptomic runs.

Supplemental Table 5. Summarized QC results for Dataset 1.

Supplemental Table 6. Pseudobulk DE gene analysis of Xenium Dataset 1 comparing colon biopsies in UC PRE versus HC. Significance was set as log2fc >0.4 or <-0.4, p-adj < 0.1 and baseMean >500.

Supplemental Table 7. Pseudobulk DE gene analysis of CosMx Dataset 1 comparing colon biopsies in UC PRE versus HC. Significance was set as log2fc >0.4 or <-0.4, p-adj < 0.1 and baseMean >400.

Supplemental Table 8. Pseudobulk DE gene analysis on Dataset 3 comparing colon biopsies in ICI versus HC. Significance was set as log2fc >0.4 or <-0.4, p-adj < 0.1 and baseMean >500.

Supplemental Table 9. Pseudobulk DE gene analysis on Dataset 3 comparing colon biopsies in ICI versus UC PRE. Significance was set as log2fc >0.4 or <-0.4, p-adj < 0.1 and baseMean >500.

Supplemental Table 10. Pseudobulk DE gene analysis of Xenium Dataset 1 comparing colon biopsies in UC PRE Non-Responders versus UC PRE Responders. Significance was set as log2fc >0.4 or <-0.4, p-adj < 0.1 and baseMean >500.

Supplemental Table 11. iSCST gene signatures used for Gene Set Enrichment Analysis (GSEA). Numeric ID, Affymetrix numeric probe identifier corresponding to each gene.
